# Randomized trial assessing impact of probiotic supplementation on gut microbiome and clinical outcome from targeted therapy in metastatic renal cell carcinoma

**DOI:** 10.1002/cam4.3569

**Published:** 2020-11-01

**Authors:** Nazli Dizman, JoAnn Hsu, Paulo G. Bergerot, John D. Gillece, Megan Folkerts, Lauren Reining, Jeffrey Trent, Sarah K. Highlander, Sumanta K. Pal

**Affiliations:** ^1^ Department of Medical Oncology and Experimental Therapeutics City of Hope Comprehensive Cancer Center Duarte CA USA; ^2^ Department of Internal Medicine Yale New Haven Hospital, Yale University Medical School New Haven CT USA; ^3^ Pathogen and Microbiome Division Translational Genomics Research Institute North Flagstaff AZ USA; ^4^ Translational Genomics Research Institute Phoenix AZ USA

**Keywords:** dietary supplement, microbiome, probiotics, renal cell carcinoma, targeted therapies, VEGF‐TKI

## Abstract

Studies suggest a link between the gut microbiome and metastatic renal cell carcinoma (mRCC) outcomes, including evidence that mRCC patients possess a lower abundance of *Bifidobacterium* spp. compared to healthy adults. We sought to assess if a *Bifidobacterium*‐containing yogurt product could modulate the gut microbiome and clinical outcome from vascular endothelial growth factor‐tyrosine kinase inhibitors (VEGF‐TKIs). mRCC patients initiating VEGF‐TKIs, regardless of the line of therapy, were randomized to probiotic‐supplemented (two 4 oz. servings of the probiotic yogurt product daily) or probiotic‐restricted arms. Stool samples were collected prior to therapy and at weeks 2, 3, 4, and 12. Microbiome composition was assessed using whole‐metagenome sequencing. A total of 20 patients were randomized. *Bifidobacterium animalis*, the active ingredient of the probiotic supplement, reached detectable levels in all patients in the probiotic‐supplemented arm versus two patients in the probiotic‐restricted arm. Clinical benefit rate was similar in probiotic‐supplemented versus probiotic‐restricted arms (70% vs. 80%, *p* = 0.606). Linear discriminant analysis (LDA) effect size analysis of MetaPhIAn2 abundance data predicted 25 enriched species demonstrating an LDA score >3 in either clinical benefit or no clinical benefit. In patients with clinical benefit (vs. no clinical benefit), *Barnesiella intestinihominis* and *Akkermansia muciniphila* were significantly more abundant (*p* = 7.4 × 10^−6^ and *p* = 5.6 × 10^−3^, respectively). This is the first prospective randomized study demonstrating modulation of the gut microbiome with a probiotic in mRCC. Probiotic supplementation successfully increased the *Bifidobacterium* spp. levels. Analysis of longitudinal stool specimens identified an association between *B. intestinihominis*, *A. muciniphila*, and clinical benefit with therapy.

**Trial Registration**: NCT02944617

## Graphical abstract

Our study provides novel evidence regarding gut microbiome modulation with a *Bifidobacterium*‐containing yogurt in metastatic renal cell carcinoma patients receiving targeted therapies and specifies the microbiota members (*Barnesiella intestinihominis*, *Akkermansia muciniphila*) associated with clinical benefit from targeted therapies in mRCC.

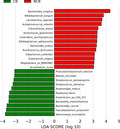

## 1 INTRODUCTION

Treatment of metastatic renal cell carcinoma (mRCC) has evolved rapidly over the past two decades, initially with the advent of targeted therapy and more recently with the introduction of checkpoint inhibitors.^1^ Across lines of therapy for advanced disease, there is a debate as to whether targeted therapy or checkpoint inhibition represents an optimal approach. Several studies have performed detailed genomic assessments of patients receiving immunotherapy, revealing alterations in specific genes (e.g., *PBRM1*) or genomic signatures that can predict response.^2^, ^3^, ^4^ However, at present, there is no biomarker‐based approach to treatment selection for mRCC.

Several groups have looked to the gut microbiome as a potential modulator of immune therapy response. They have found putative associations between microbiome composition and clinical benefit (either response rate [RR] or progression‐free survival [PFS]) with immunotherapy.^5^, ^6^, ^7^ These studies encompass multiple malignancies, including melanoma, non‐small cell lung cancer (NSCLC), and RCC. In RCC, Routy et al. specifically demonstrated an association between levels of *Akkermansia* spp. and several other bacterial species with clinical benefit.^5^ The link between the microbiome and clinical benefit in mRCC was further corroborated by reports from Derosa et al. suggesting that antibiotic therapy could profoundly alter RR and PFS observed with immunotherapy.^8^

A lesser studied phenomenon is the link between the microbiome and targeted therapy, which remains a mainstay of treatment for mRCC. The most frequently employed targeted therapies for mRCC abrogate signaling through the vascular endothelial growth factor (VEGF) pathway. At present, six VEGF‐tyrosine kinase inhibitors (VEGF‐TKIs) are approved by the United States Food and Drug Administration (US FDA). These are axitinib, sorafenib, sunitinib, pazopanib, lenvatinib/everolimus, and cabozantinib. We have previously reported that certain microbiome members (specifically, *Prevotella* spp. and *Bacteroides* spp.) can influence the rate of diarrhea associated with these therapies. In addition, our findings suggested that the relative abundance of *Bifidobacterium* spp. was lower in patients with mRCC compared to the historical studies of healthy subjects.^9^ A limitation of our study (similar to the aforementioned trials) included retrospective capture of clinical data. Furthermore, no studies have assessed whether modulation of the microbiome can impact clinical outcome. To address this, we conducted a prospective, randomized trial to determine if a probiotic supplement could modulate the clinical outcome among patients receiving standard‐of‐care VEGF‐TKI therapy.

## 2 PATIENTS AND METHODS

### 2.1 Patient selection

Key eligibility for this study included a pathologically verified diagnosis of RCC, metastatic disease by standard criteria (AJCC 7th edition, 2010), and planned treatment with a VEGF‐TKI therapy indicated for mRCC by the US FDA.^10^ Patients had to express an intent to comply with study related procedures, including intake of the probiotic supplement and submission of stool specimens at predefined timepoints (both defined subsequently). Patients with known intolerance to lactose or other constituents of the probiotic supplement were excluded, as were patients taking antibiotics or those with a perceived indication for antibiotic therapy. In addition, patients with irritable bowel syndrome, Crohn's disease, or other clinically significant gastrointestinal conditions that might confound the assessment of the VEGF‐TKI‐related diarrhea endpoint were excluded.

The written consent form and the protocol were approved by the City of Hope Institutional Review Board, scientific review committee and data safety monitoring board. All patients enrolled and evaluated on the study provided written informed consent. The study was conducted in accordance with the amended Declaration of Helsinki and the International Conference on Harmonization Guidelines.

### 2.2 Study design

The study was conducted using an open‐label, randomized design evaluating change in baseline *Bifidobacterium* spp. abundance between the probiotic‐supplemented group and the group without probiotic supplementation as the primary endpoint. If patients were randomized to the probiotic‐supplemented group, they were asked to purchase the yogurt product. Patients consumed a 4‐oz‐serving of probiotic yogurt twice daily for 3 months. Patients on both arms were cautioned not consume other yogurt or yogurt‐containing foods and were asked to refrain from using other probiotic supplements during the 3‐month study period. A computerized simple randomization process was employed. Notably, methods of dietary intervention in different arms (i.e., recommendation of a dietary intervention without direct supply of actual food contents) replicated the format of a large prospective study published in the New England Journal of Medicine assessing the cardiac benefit of a Mediterranean diet.^11^

### 2.3 Biomarker assessment

Patients were asked to submit fecal material in a 100 mL collection container, which was stored in a cooled transfer container. A detailed standard operating procedure (SOP) pamphlet was generated and shared with patients (see Documents S1––Study Protocol). Samples were collected by participants at home and dropped off at a FedEx location on the day of sample collection. Collection occurred at pretreatment, week 2, week 3, week 4, and week 13 timepoints, relative to initiation of VEGF‐TKI therapy. Participants who stopped taking VEGF‐TKI prior to week 13 had a final sample collected within a week of discontinuation.

Gut microbiota composition was assessed using whole‐genome shotgun metagenomic sequencing, using previously published methods.^12^ Briefly, DNA was extracted from stool samples using the MagMax PowerMicrobiome extraction kit with the KingFisher Flex magnetic purification system (Thermo Fisher). DNAs were quantitated by Qubit fluorometer assay (Thermo Fisher) and sequencing libraries were generated using the KAPA Biosystems Hyper Prep Kit (KK8504; Roche). Libraries were quantified using a KAPA Library Quantification Kit (KR0405, Roche), were pooled and then, sequenced on the Illumina NextSeq platform to an average depth of 2 Gb per sample.

Demultiplexed reads were quality trimmed using Trimmomatic to remove adapters and low‐quality bases and reads.^13^ Trimmed metagenomic reads were taxonomically profiled using MetaPhlAn 2.0.^14^, ^15^

### 2.4 Statistical analysis

Twenty patients were planned for enrollment in this pilot study. Comparison of categorical variables such as rate of diarrhea and clinical benefit from therapies across arms was performed using Fishers' exact test. Survival estimates were calculated using Kaplan–Meier method. In addition to the prespecified endpoints of our study protocol, we have performed Linear discriminant analysis (LDA) effect size (LEfSe) to identify taxa that were significantly different in metagenomes of patients with clinical benefit, a best response of either complete/partial response or stable disease for over 6 months, and no clinical benefit, a best response of progressive disease.^14^

## 3 RESULTS

### 3.1 Patient characteristics

Between December 2017 and September 2019, 21 patients were enrolled and randomized. For the current analysis, 20 patients were deemed evaluable––one patient was excluded because of early death 12 days into receipt of systemic therapy due to rapid disease progression (Figure S1). Of the 20 evaluated patients, 15 (75%) were male and 5 (25%) were female with a median age of 67.5 (range, 32–81). Median lines of VEGF‐TKI therapy were 2 (range, 1–6). The most common VEGF‐TKIs rendered were cabozantinib (45%), sunitinib (30%), and lenvatinib/everolimus (20%). Demographic criteria based on treatment arm is presented in Table 1.

**TABLE 1:** Patient characteristics and clinical outcomes

	Overall (*n* = 20)	Probiotic supplemented (*n* = 10)	Probiotic restricted (*n* = 10)	*p* value
Baseline patient characteristics				
Age, median (range)	67.5 (32–81)	67.0 (57–81)	67.5 (32–78)	0.850
Gender				
Male	15 (60%)	8 (80%)	7 (70%)	0.615
Female	5 (40%)	2 (20%)	3 (30%)	
Histology				
Clear cell RCC	16 (80%)	8 (80%)	8 (80%)	0.709
Non‐clear cell RCC	4 (20%)	2 (20%)	2 (20%)	
Papillary RCC	3 (15%)	2 (20%)	1 (10%)	
Sarcomatoid RCC	1 (5%)	—	1 (10%)	
IMDC risk category				
Favorable	7 (35%)	4 (40%)	3 (30%)	0.638
Intermediate	11 (55%)	5 (50%)	6 (60%)	
Poor	2 (10%)	1 (10%)	1 (10%)	
VEGF‐TKI				
Cabozantinib	9 (45%)	7 (70%)	2 (20%)	0.166
Sunitinib	6 (30%)	2 (20%)	4 (40%)	
Lenvatinib/Everolimus	4 (20%)	1 (10%)	3 (30%)	
Axitinib	1 (5%)	—	1 (10%)	
Line of therapy, median (range)	2 (1–6)	3 (1–6)	2 (1–3)	0.027
Line of therapy				
First‐line	6 (30%)	2 (20%)	4 (40%)	0.650
Second‐line	6 (30%)	1 (10%)	5 (50%)	
Third‐line	6 (30%)	5 (50%)	1 (10%)	
Further lines	2 (10%)	2 (20%)	0 (0%)	
Clinical outcomes				
Best response				
Partial response	3 (30%)	2 (20%)	1 (10%)	0.392
Stable disease	12 (60%)	5 (50%)	7 (70%)	
Progressive disease	3 (30%)	1 (10%)	2 (20%)	
N/E	2 (20%)	2 (20%)	0 (0%)	
Clinical benefit rate	75%	70%	80%	0.606
Progression‐free survival, months, and median (95% CI)	10.8 (5.3–16.4)	6.2 (2.2–10.3)	13.8 (6.2–21.5)	0.077
VEGF‐TKI stopped	14 (70%)	7 (70%)	7 (70%)	0.639
Progression	10 (50%)	3 (30%)	7 (70%)	
Toxicity	4 (20%)	4 (40%)	0 (0%)	
VEGF‐TKI continues	6 (30%)	3 (30%)	3 (30%)	
Diarrhea, present	8 (40%)	4 (40%)	4 (40%)	1.000
Grade 1–2	8 (40%)	4 (100%)	4 (100%)	
Grade 3–4	0 (0%)	0 (0%)	0 (0%)	
Diarrhea, absent	12 (60%)	6 (60%)	6 (60%)	

Abbreviations: IMDC, International Metastatic Renal Cell Carcinoma Consortium; N/E, not evaluable; VEGF‐TKI, vascular endothelial growth factor tyrosine kinase inhibitor.

### 3.2 Characterization of microbiota

Whole‐metagenome sequencing was performed on stool collected from all 20 patients, including specimens collected at baseline (prior to receipt of VEGF‐TKI therapy) and at several sequential timepoints thereafter (see Section 2). With respect to the intervention, we detected *Bifidobacterium animalis* (the active ingredient of the probiotic yogurt product) in 67% of post‐baseline specimens in patients in the probiotic‐supplemented group, as compared to 0.023% of post‐baseline specimens in the probiotic‐restricted group.

### 3.3 Clinical response and toxicity

Among those evaluable for response, three patients (15%) achieved a partial response and 12 patients (60%) achieved stable disease as a best response. Three patients (15%) had progressive disease. Response based on treatment arm is presented in Table 1; no significant difference was seen in response based on treatment arm. Median PFS was 10.8 months (95% CI, 5.3–16.4). Diarrhea was reported in eight evaluable patients (40%), with none reporting grade 3/4 diarrhea by CTCAE v4.0 criteria. There was no significant difference in diarrhea incidence based on treatment arm (Table 1).

### 3.4 Clinical response and microbiota

**FIGURE 1:**
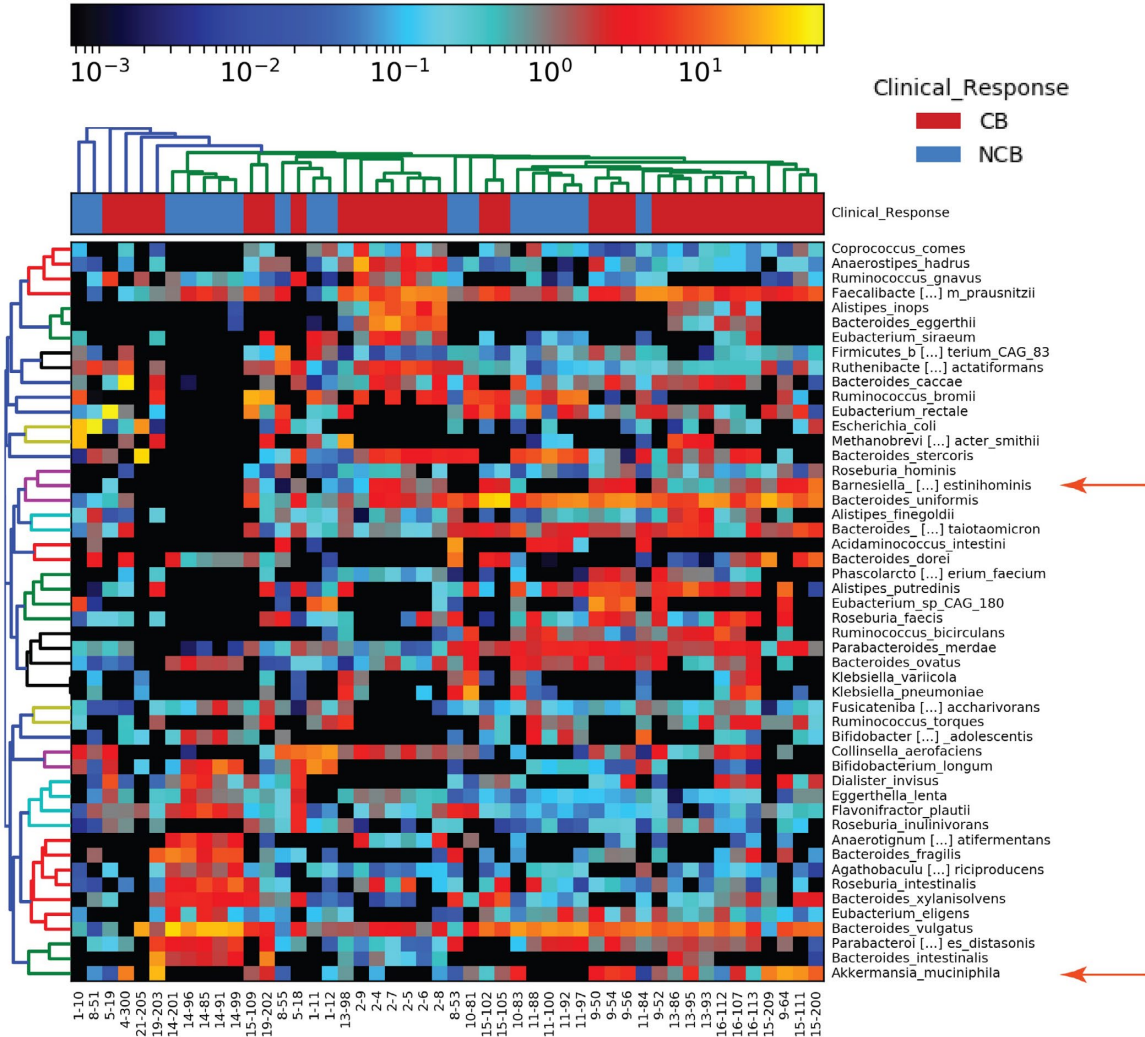
Hierarchical cluster of top 20 taxa identified in all metagenomic samples identified by MetaPhlAn2.^14^, ^15^ Bray–Curtis clustering and heatmap generation were performed using hclust2. Clinical benefit by patient is indicated at the top by color boxes. *Barnesiella intestinihominis* and *Akkermansia muciniphila* are indicated with red arrows. Patient number and sample number are shown on the x‐axis

Bray–Curtis‐based hierarchical clustering revealed differences between patients with clinical benefit and no clinical benefit (Figure 1). Most clustering was by patient, for example, patients 14, 2, 15, and 9 from right to left on the x‐axis, however there was larger cluster on the right of the heatmap that included samples from four patients (9, 13, 15, and 16). Of note was the presence of *Akkermansia muciniphila*, *Bacteroides caccae*, and *Faecalibacterum prausnitzii* in the majority of samples from patients achieving clinical benefit. *Barnesiella intestinihominis*, a member of the family Porphyromonadaceae, was almost exclusively present in those who achieved clinical benefit.

**FIGURE 2:**
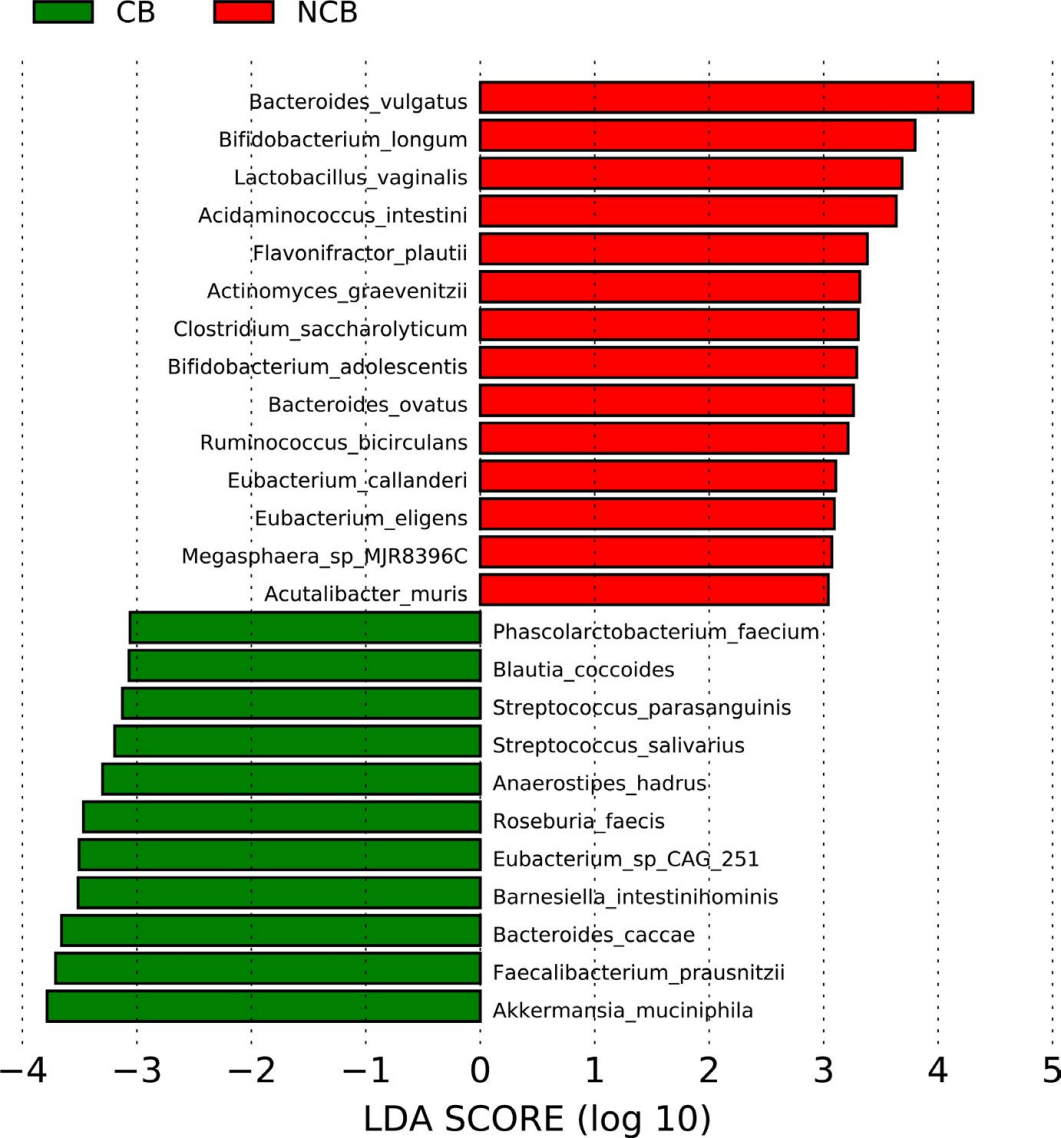
LEfSe plot of bacterial taxa with linear discriminant analysis (LDA) scores greater than three significant associated with no clinical benefit (NCB, red) and clinical benefit (CB, green). LDA score (log 10) is shown on the x‐axis

**FIGURE 3:**
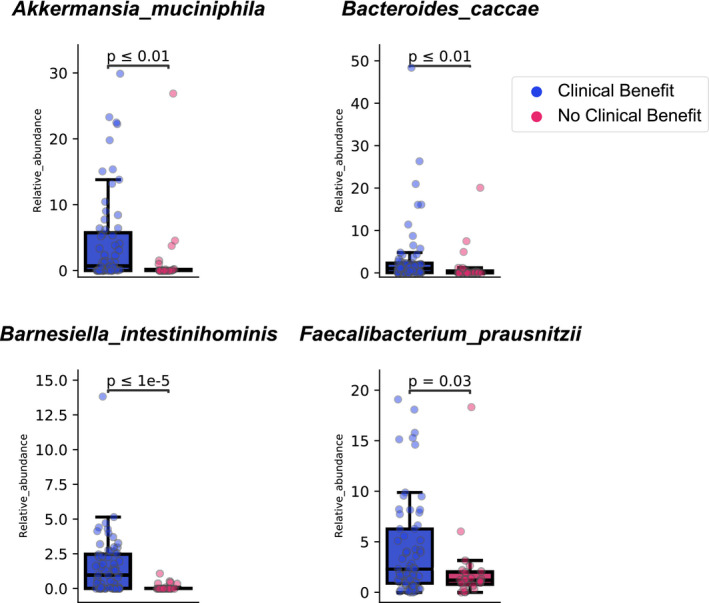
Relative abundances of *Akkermansia muciniphila*, *Bacteroides caccae*, *Faecalibacterum prausnitzii*, and *Barnesiella intestinihominis* in patients who has clinical benefit and no clinical benefit with VEGF‐TKIs. Boxplots were generated using the Kruskal–Wallis H test

Linear discriminant effect size (LEfSe) identified 25 species that discriminated between the two study outcomes with an LDA score greater than three (Figure 2).^14^ *B. intestinihominis* was in this group and had the lowest *p* value (7.4 × 10^−6^) of all organisms in the clinical benefit category. Similarly, *Akkermansia muciniphila* was more abundant in the clinical benefit group (*p* = 5.6 × 10^−3^). *Bacteroides caccae* was also a discriminator in the clinical benefit group (*p* = 8.5 × 10^−3^). Figure 3 shows relative abundance of *A. muciniphila*, *B. caccae*, *F. prausnitzii*, and *B. intestinihominis* in clinical benefit and no clinical benefit groups. In each case, the relative abundance between treatment arms was significant. Somewhat surprisingly, *Bifidobacterium longum*, a species also used as a probiotic, was a significant contributor to no clinical benefit (*p* = 3 × 10^−3^). No difference in Shannon diversity index based on clinical benefit nor progression‐free survival was observed (data not shown).

## 4 DISCUSSION

To the best of our knowledge, the current study is the first to assess modulation of the microbiome to alter clinical outcome in patients with mRCC. The study used a commonly recommended probiotic yogurt which has been evaluated in randomized trials for gastrointestinal ailments such as constipation.^16^ While preclinical studies have identified a potential anticancer effect of this probiotic yogurt in murine models of colon cancer, we failed to show any difference across arms in regard to cancer progression or gastrointestinal toxicity.^17^ Longitudinal stool microbiome profiling showed *B. animalis*, the key component of the probiotic yogurt supplement, was prevalent in the probiotic‐supplemented arm but nearly absent in the probiotic‐restricted arm. This finding suggests the feasibility of microbiome modulation through dietary interventions and also affirms the literature reporting the transient and dose‐ and duration‐dependent nature of the impacts of diet on gut microbiome.^18^, ^19^, ^20^ Sequencing revealed the first reported association between a specific stool microbial species (*B. intestinihominis*) and clinical benefit from VEGF‐TKIs.

As noted, previous studies have defined a link between clinical benefit and response to immunotherapy in patients with elevated levels of stool *Akkermansia* spp.^5^ We also observed an association between *A. muciniphila* and clinical benefit from targeted therapies that might suggest that the relationship between the abundance of *A. muciniphila* and improved clinical outcomes may be prognostic in nature rather than predictive. Furthermore, the significance of the association between clinical benefit and *A. muciniphila* (*p* = 0.0056) was not as great as that calculated for *B. intestinihominis*. We also observed a significant increase in *B. caccae* and *F. prausnitzii* in the clinical benefit group. *B. caccae* and *F. prausnitzii* have been associated with positive response to immune checkpoint therapy in melanoma.^21^ As opposed to a direct antitumor effect, preclinical models accompanying these studies suggest a complex interplay in which these bacteria affect T‐cell trafficking. *B. intestinihominis* may have distinct immunomodulatory properties. In murine cancer models treated with cyclophosphamide, *B. intestinihominis* increased in abundance within the colon during treatment.^22^ This in turn led to an increased concentration of interferon‐α producing γδ‐T‐cells within the gut. In patients with ovarian and lung cancer receiving chemoimmunotherapy, *B. intestinihominis* within the gut was associated with longer PFS and OS. It is unclear if VEGF‐TKI therapy elicits the same impact––preclinical studies to evaluate this phenomenon are planned.

Limitations of the study include the small sample size and the heterogeneity of their baseline clinical characteristics, treatment types, and lines. The potential impact of this limitation on the generalization of clinical outcomes beyond our sample should be cautious in nature. Although an expansion of the study was planned, accrual was very slow with 21 patients randomized over 2 years. Barriers to accrual included the stool collection kit––the kit designed at the start of study implementation was larger and onerous for patients to collect and submit. To ensure consistency across specimens evaluated in the current study, this kit was maintained through the course of the study. More recent microbiome‐directed trials that we have initiated, however, make use of a more compact kit.^23^ The updated kit also includes a preservative solution in which stool is stored that might mitigate changes evolving from the time between specimen collection and analysis. The need for strict compliance was also a barrier to accrual in the current study––many patients were unwilling to (a) either consume a yogurt‐based supplement daily or (b) unwilling to forgo taking such a product. Data from food diaries maintained by patients in the current study, however, indicate minimal issues with compliance, with all patients randomized to probiotic therapy reporting intake of the probiotic‐supplemented yogurt in over 99% of the days while on study and 100% of patients randomized to the control arm taking no yogurt‐based products for the 3‐month study period.

## 5 CONCLUSIONS

Our randomized, prospective study demonstrated that dietary interventions result in modulation of gut microbiome in patients with metastatic renal cell carcinoma receiving VEGF‐TKI therapy. In addition, this study is the first to suggest that components of the stool microbiome, *A. muciniphila* and *B. intestinihominis*, may predict clinical benefit in patients with mRCC receiving VEGF‐TKI therapy, distinct from previously reported predictors of checkpoint inhibitor response. We also demonstrate proof of principle, with the active ingredient of our probiotic supplement detectable specifically in patients randomized to receive it. Confirmation of our findings in larger series is warranted.

## CONFLICT OF INTEREST

SKP reports consulting roles in Genentech, Aveo, Eisai, Roche, Pfizer, Novartis, Exelixis, Ipsen, BMS, and Astellas. ND has consulting roles in Vivreon. JDG, MF, LR, SH, and JT are employees of Translational Genomics Research Institute, Flagstaff, AZ. JH and PB declare no conflict of interest.

## Supporting information

Fig S1

Supplementary Material

## Data Availability

Datasets generated and/or analyzed during the current study are not publicly available since data sharing has not been included in the institutional review board approval.
