# Supplementary material for: Randomized trial assessing impact of probiotic supplementation on gut microbiome and clinical outcome from targeted therapy in metastatic renal cell carcinoma
Source: Cancer Med. 2020 Nov 1;10(1):79–86. doi: 10.1002/cam4.3569 (PMC7826461; doi:10.1002/cam4.3569)

Supplemental Figure 1. CONSORT flow diagram detailing the inclusion and exclusion of the participants in the study

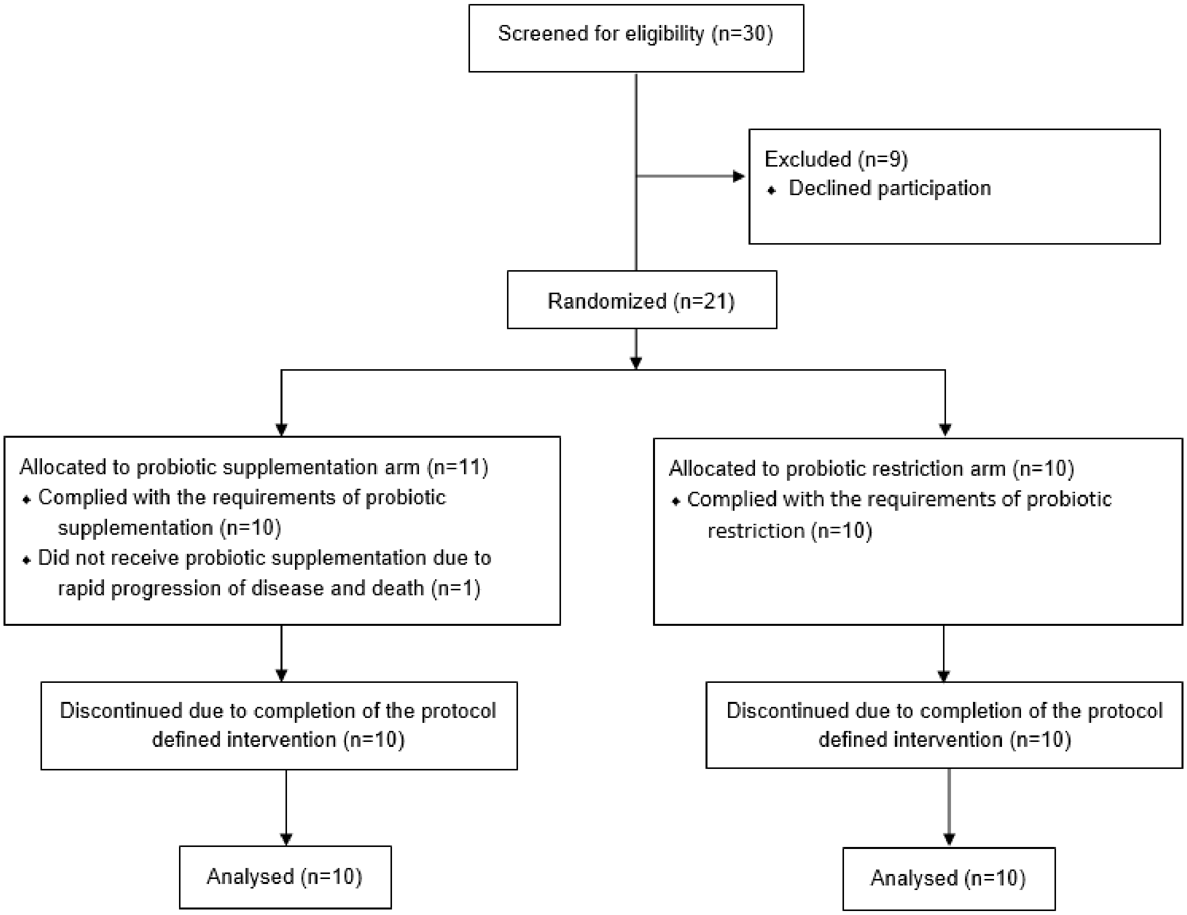

Supplement: Supplementary file 1 — Fig S1 [file CAM4-10-79-s001.pdf]
