# Supplementary material for: Randomized trial assessing impact of probiotic supplementation on gut microbiome and clinical outcome from targeted therapy in metastatic renal cell carcinoma
Source: Cancer Med. 2020 Nov 1;10(1):79–86. doi: 10.1002/cam4.3569 (PMC7826461; doi:10.1002/cam4.3569)
Supplement: Supplementary file 2 — Supplementary Material [file CAM4-10-79-s002.pdf]

CITY OF HOPE NATIONAL MEDICAL CENTER
1500 E. DUARTE ROAD
DUARTE, CA 91010

DEPARTMENT OF MEDICAL ONCOLOGY AND THERAPEUTICS RESEARCH

TITLE: Assessment of the role of Bifidobacterium-containing food supplement Activia™ and Bacteriomic Profiling and Other Biomarkers Associated with Vascular Endothelial Growth Factor Tyrosine Kinase Inhibitor (VEGF-TKI)-Induced Diarrhea in Patients with Metastatic Renal Cell Carcinoma (mRCC)

CITY OF HOPE PROTOCOL NUMBER: 16088 **VERSION:** 09

Initial Submission	Protocol dated 06/27/2016	Version 00
Amendment 1	Protocol dated 05/25/2017	Version 01
Amendment 2	Title page dated 01/31/2018	Version 02
Amendment 3	Title page and Protocol dated 02/28/2018	Version 03
Amendment 4	Title page and Protocol dated 10/30/2018	Version 04
Amendment 5	Title page and Protocol dated 12/14/2018	Version 05
Amendment 6	Title page and Protocol dated 02/25/2019	Version 06
Amendment 7	Title page dated 06/25/19	Version 07
Amendment 8	Title page and Protocol dated 06/26/2019	Version 08
Amendment 9	Title page and Protocol dated 06/12/2020	Version 09

SPONSOR/IND NUMBER: City of Hope / N/A

DISEASE SITE: Renal cell carcinoma

STAGE (if applicable): N/A

MODALITY: N/A

PHASE/TYPE: Non-therapeutic / Prospective Sample Collection

PRINCIPAL INVESTIGATOR:
Sumanta Pal, M.D.
City of Hope Comprehensive Cancer Center
1500 E. Duarte Rd
Duarte, CA 91010
P: 626-256-4673
F: 626-301-8233
E-mail: spal@coh.org

COLLABORATING INVESTIGATORS:
Clayton Lau, M.D.
Marcin Kortylewski, Ph.D.
Huiqing Wu, M.D.
Ravi Salgia, M.D.
Tanya Dorff, M.D.
Sarah Highlander, PhD at TGen

STUDY STATISTICIANS:
Paul Frankel, Ph.D.
Sierra Min Li, Ph.D.

PARTICIPATING SITES: City of Hope, Duarte, CA

FUNDING SUPPORT: Pfizer, Inc

CITY OF HOPE NATIONAL MEDICAL CENTER
1500 E. DUARTE ROAD
DUARTE, CA 91010

DEPARTMENT OF MEDICAL ONCOLOGY AND THERAPEUTICS RESEARCH

TITLE: Assessment of the role of Bifidobacterium-containing food supplement Activia™ and Bacteriomic Profiling and Other Biomarkers Associated with Vascular Endothelial Growth Factor Tyrosine Kinase Inhibitor (VEGF-TKI)-Induced Diarrhea in Patients with Metastatic Renal Cell Carcinoma (mRCC)

CITY OF HOPE PROTOCOL NUMBER: 16088 **VERSION:** 09

Initial Submission	Protocol dated 06/27/2016	Version 00
Amendment 1	Protocol dated 05/25/2017	Version 01
Amendment 2	Title page dated 01/31/2018	Version 02
Amendment 3	Title page and Protocol dated 02/28/2018	Version 03
Amendment 4	Title page and Protocol dated 10/30/2018	Version 04
Amendment 5	Title page and Protocol dated 12/14/2018	Version 05
Amendment 6	Title page and Protocol dated 02/25/2019	Version 06
Amendment 7	Title page dated 06/25/19	Version 07
Amendment 8	Title page and Protocol dated 06/26/2019	Version 08
Amendment 9	Title page and Protocol dated 06/12/2020	Version 09

SPONSOR/IND NUMBER: City of Hope / N/A

DISEASE SITE: Renal cell carcinoma

STAGE (if applicable): N/A

MODALITY: N/A

PHASE/TYPE: Non-therapeutic / Prospective Sample Collection

PRINCIPAL INVESTIGATOR:
Sumanta Pal, M.D.
City of Hope Comprehensive Cancer Center
1500 E. Duarte Rd
Duarte, CA 91010
P: 626-256-4673
F: 626-301-8233
E-mail: spal@coh.org

COLLABORATING INVESTIGATORS:
Clayton Lau, M.D.
Marcin Kortylewski, Ph.D.
Huiqing Wu, M.D.
Ravi Salgia, M.D.
Tanya Dorff, M.D.
Sarah Highlander, PhD at TGen

STUDY STATISTICIANS:
Paul Frankel, Ph.D.
Sierra Min Li, Ph.D.

PARTICIPATING SITES: City of Hope, Duarte, CA

FUNDING SUPPORT: Pfizer, Inc

EXPERIMENTAL DESIGN SCHEMA

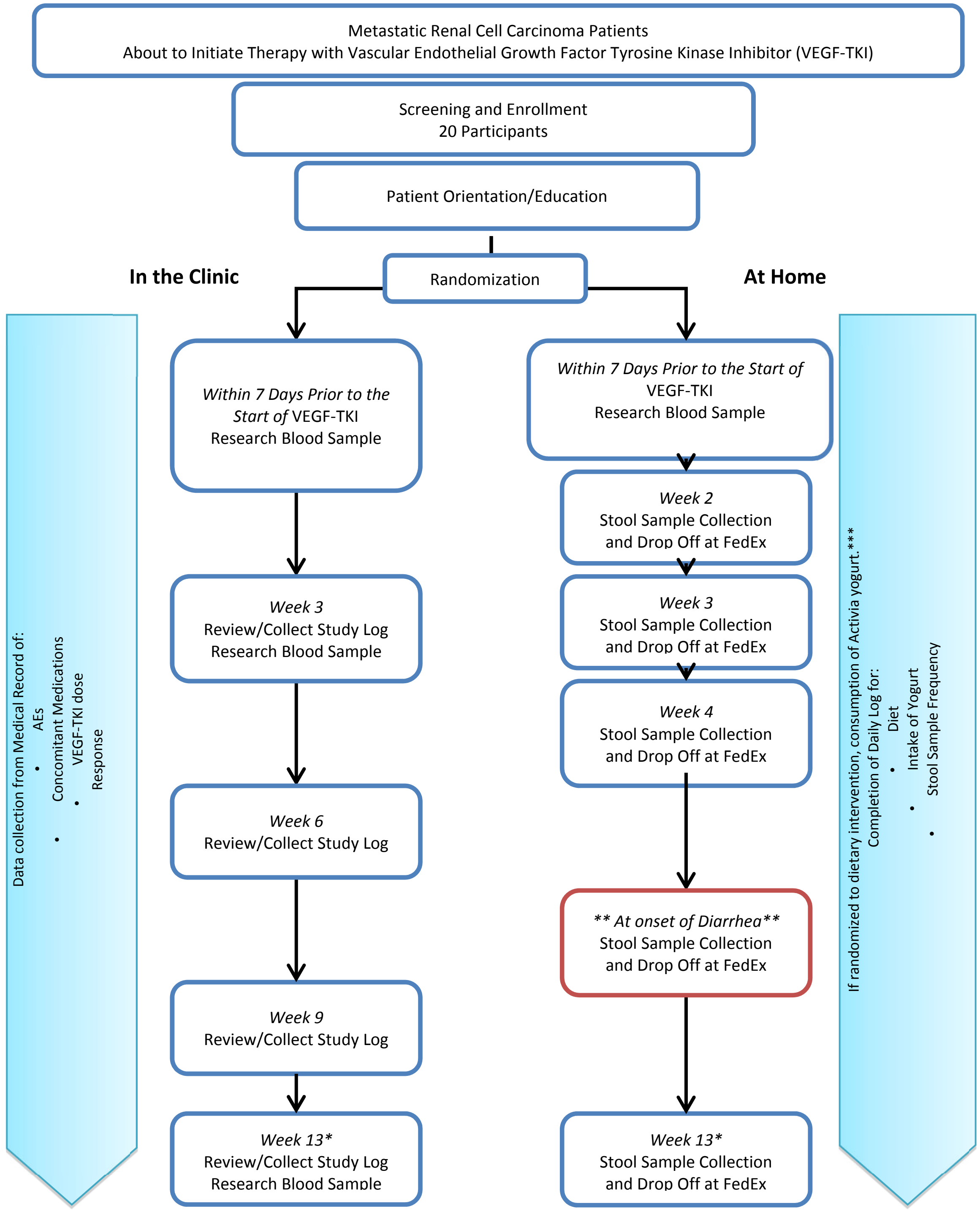

***If randomized to dietary intervention, participant will be asked to consume one 4 oz serving of Activia vanilla yogurt in the morning and one 4 oz serving of Activia vanilla yogurt in the evening for the duration of the study.
**Within 24 hours of onset of diarrhea following initiation of VEGF-TKI, a stool sample is to be provided
*If a participant ends VEGF-TKI before week 13, a final sample is to be provided, and the study logs to that time point are to be collected. Study participation will end at that time.

TABLE OF CONTENTS

SECTION		PAGE
Experimental Design Schema		3
Table of Contents		4
Abbreviations		5
1.0	Objectives	7
2.0	Background and rationale	7
2.1	Introduction and Rationale for Study	7
2.2	Overview and rationale of study design	10
3.0	Participant Number, Cohort Characteristics, Eligibility, and Involvement	11
3.1	Sample number and cohort characteristics	11
3.2	Eligibility	11
3.3	Duration of Participation and Overview of What Is Expected From Participants	12
4.0	Study Activity Table	13
4.1	Study Activity Table	13
5.0	Clinical Data Collection	14
5.1	Participant Daily Diary	14
5.2	Data Collected from the Medical Record	14
6.0	Blood Specimens, assessment of Treg, STAT3 and MDSC	14
6.1	Research Blood Specimen Collection and Transfer to Processing Laboratory	14
6.2	Initial Sample Processing – Isolation of PBMCs	15
6.3	Sample Analysis	15
7.0	Dietary intervention, Stool Specimens, Bacteriomic Profiling	15
7.1	Dietary Intervention	15
7.2	Stool Specimen Collection	16
7.3	Laboratory Processing and Analysis	16
8.0	Statistical Considerations	17
8.1	Study Design and Statistical Analysis	18
8.2	Sample Size and Projected Accrual Rate and Study Duration	19
9.0	Human Subject Issues	19
9.1	Recruitment of Subjects	19
9.2	Advertisements	19
9.3	Informed Consent	19
9.4	Participant Withdrawal from Research	20
9.5	Study location and Performance Sites	20

9.6 Potential Risks and Benefits to Participation 20
9.7 Alternatives to Participation 20
9.8 Financial Obligations and Compensation 21
9.9 Registration into MIDAS 21
9.10 Confidentiality 21
10.0 Data and Safety Monitoring, Unanticipated Problems and Adverse Event Reporting 21
10.1 Definition of Risk Level 21
10.2 Monitoring and Personnel Responsible for Monitoring 21
10.3 Unanticipated Problems (UP) Involving Risks to Subjects or Others 21
10.4 Deviations 22
10.5 Reporting Deviations 22
10.6 Single Subject Exception (SSE) Amendment Request 22
11.0 Institutional Review Board 22
11.1 Institutional Review Board 22
12.0 References 22
Appendix A: Stool Collection Procedure 24
Appendix B: Diet and Stool Frequency Log 28
Appendix C: At Home Sample Collection Kit Contents 31
Appendix D: Yogurt-Intake Log....28
Appendix E: PHQ-9....34
Appendix F: GAD-7....35

ABBREVIATIONS

Abbreviation	Meaning
AE	Adverse event
CFR	Code of Federal Regulations
COH	City of Hope
CRA	Clinical research associate
CRF	Case report form
CTCAE	Common Terminology Criteria for Adverse Events
DSMC	Data Safety Monitoring Committee
FDA	Food and Drug Administration
GAD-7	Generalized Anxiety Disorder
GCP	Good clinical practice
ICF	Informed consent form
IRB	Institutional review board
MDSC	Myeloid-derived suppressor cells
NCI	National Cancer Institute
PBMC	Peripheral blood mononuclear cells
PHQ-9	Patient Health Questionnaire
PI	Principal investigator
PMT	Protocol monitoring team

RPN	Research patient number
SAE	Serious adverse event

1.0 OBJECTIVES

Primary objective

- To determine if adding Activia™ yogurt (containing Bifidobacterium lactis DN-173 010) to the diet of patients with metastatic renal cell carcinoma (mRCC) increases the level of Bifidobacterium spp in stool.

Secondary objectives

- To determine if Activia™ reduces the incidence of diarrhea for mRCC patients treated with vascular endothelial growth factor – tyrosine kinase inhibitor (VEGF-TKI) therapy.
- To compare pre-treatment levels of circulating Treg cells and levels of peripheral STAT3 in patients with and without diarrhea with VEGF-TKI therapy, and correlate with Bifidobacterium, other bacteria, and Activia™.
- To determine if patients with the diarrhea in mRCC patients treated with VEGF-TKI therapy have a lower baseline level of *Bifidobacterium spp.*
- To assess the change in global stool microbiome profile of patients receiving therapy with VEGF-TKI therapy with or without Activia™
- To better assess the feasibility of stool collection and bacteriomic profiling.
- To explore association between psychosocial symptoms (anxiety and depression) and bacteriomic profile and gastrointestinal toxicity in mRCC patients receiving VEGF-TKI therapy.

2.0 BACKGROUND AND RATIONALE

2.1 Introduction and Rationale for Study

Recent Developments in mRCC: A Crowded Landscape

Over the past 6 years, a total of 6 targeted therapies have been approved for the treatment of mRCC.[1] These agents can be divided into two principal categories: (1) inhibitors of the vascular endothelial growth factor receptor (VEGFR), and (2) inhibitors of the mammalian target of rapamycin (mTOR). The former category includes both VEGF-TKIs such as axitinib, sunitinib, pazopanib and sorafenib, as well as monoclonal antibodies such as bevacizumab that inhibit VEGFR through ligand binding. National Comprehensive Cancer Network (NCCN) guidelines recommend use of vascular endothelial growth factor directed therapies across different lines of mRCC treatment. For example sunitinib, pazopanib, axitinib, sorafenib and bevacizumab (the latter in combination with interferon-α, IFN-α) is recommended for the first and subsequent lines of mRCC treatment and cabozantinib in the second and further lines of treatment..[2] In contrast, the two currently approved mTOR inhibitors are generally implemented in more selected settings – for instance, everolimus is used after failure of VEGF-TKI therapy, while temsirolimus is used in patients with poor-risk disease or non-clear cell histology. All together, VEGF-TKIs are the most commonly utilized treatments in patients with mRCC in the United States. [3]

Limitations to Therapy: Managing Toxicity

The multitude of available agents for mRCC often makes it challenging for the oncologist and patient to select the appropriate therapeutic strategy. For instance, for the treatment-naïve patient with mRCC, how might the oncologist choose between the monoclonal antibody bevacizumab, and VEGF-TKIs? There are no head to head comparisons between the agents, and thusly, the oncologist is virtually forced to make cross-trials comparisons. Aside from considering the efficacy of these therapies across studies, the

oncologist may consider toxicity. Diarrhea appears to be particularly problematic with VEGF-TKI therapy, for example sunitinib nearly 50% of patients reporting some degree of diarrhea, usually first occurring within the first two cycles of therapy , and 10% of patients developing grade 3/4 toxicity (i.e., toxicities warranting dose modification or treatment discontinuation).[4] In contrast, few patients experience severe diarrhea in association with bevacizumab therapy.[5,6] The phenomenon of diarrhea appears to be quite consistent across VEGF-TKIs, as seen in Table 1. Notably, even VEGF-TKIs used in the second line setting, such as axitinib, yield similar rates of diarrhea.[7] The frequency of diarrhea encountered with these agents is quite startling.

Table 1. Frequency of diarrhea reported in pivotal phase III trials assessing VEGF-TKIs for mRCC.[1,2,3]

Agent	N	Grade 1 or 2	Grade 3 or 4	Overall
Sorafenib	451	41%	2%	43%
Sunitinib	375	52%	9%	61%
Pazopanib	290	48%	4%	52%
**Cumulative**	1116	47%	5%	51%

[1]Escudier B, Eisen T, Stadler WM, et al. Sorafenib in Advanced Clear-Cell Renal-Cell Carcinoma. N Engl J Med 2007;356:125-34.
[2]Motzer RJ, Hutson TE, Tomczak P, et al. Overall Survival and Updated Results for Sunitinib Compared With Interferon Alfa in Patients With Metastatic Renal Cell Carcinoma. J Clin Oncol 2009;27:3584-90.
[3]Sternberg CN, Davis ID, Mardiak J, et al. Pazopanib in Locally Advanced or Metastatic Renal Cell Carcinoma: Results of a Randomized Phase III Trial. J Clin Oncol 2010;28:1061-8.

Bacteriomic Profiling: A Laboratory-Based Biomarker of VEGF-TKI Induced Diarrhea?

Recent technological advances now allow for complete bacteriomic profiling of intestinal flora using 16S rRNA sequencing.[8] Although such techniques have not been utilized specifically in the context of cancer therapeutics, there is existing data using fecal DNA sequencing that suggests the composition of intestinal flora can influence the development of diarrhea with specific cytotoxics. For instance, in the context of irinotecan therapy, Stringer *et al* have performed an elegant series of experiments in preclinical models suggesting that β-glucoronidase-producing *Escherichia coli* (which metabolize irinotecan) may increase during the course of irinotecan therapy.[8] The increase in *E. coli* in turn exacerbates observed gastrointestinal toxicity.

There is rationale for VEGF-TKI therapy altering the intestinal flora. Our group has previously published data suggesting an immune-mediated effect of sunitinib, decreasing T-regulatory (Treg) cell populations via STAT3.[9] Separately, it is documented that Tregs may play a role in gut homeostasis, promoting tolerance of various luminal bacteria.[10] Taken together, sunitinib and other VEGF-TKI agents in the same therapeutic class may promote an autoimmune response that shifts the equilibrium of intestinal flora composition.

Protective Effect of Probiotics

In contrast, “gastrointestinal protective” bacterial, such as *Bifidobacterium spp.*, decreased during the course of treatment. Notably, probiotic formulations including *Bifidobacterium spp.* have been noted to improve acute diarrhea in a multitude of settings.[11,12] A recently completed randomized, phase II study comparing one such probiotic to placebo in patients receiving radiotherapy for cervical cancer showed an improvement in the incidence of diarrhea with probiotic treatment.[13]

COH IRB 12096: A Pilot Study

The current team of investigators embarked on a project sponsored by the COH Cancer Center Core Grant. We collected random stool samples from a cohort of 23 patients with mRCC while receiving VEGF-directed therapies. Ultimately, 20 patients provided satisfactory samples for evaluation. Amongst these patients, the majority were treated with sunitinib or pazopanib. 16S rRNA profiling using the methods described herein was used to characterize the stool bacterial flora of these patients. Patients receiving VEGF-TKIs with mRCC **appeared to have less relative abundance of *Bifidobacterium spp* as compared to previous reports based on healthy subjects.** Patients also had higher levels of *Bacteroides spp* and lower levels of *Prevotella spp* were found in patients with diarrhea.

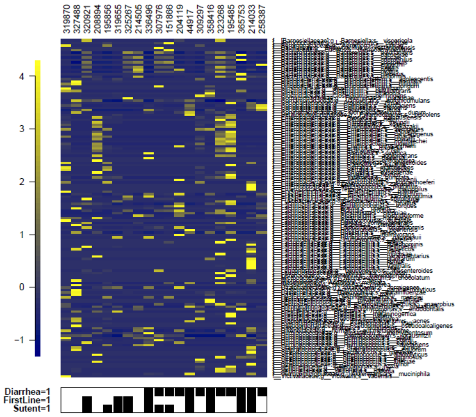

Figure 1. Stool bacterial profiling across a cohort of 20 patients with mRCC in IRB 12096 reveals two species that are more prevalent in patients without diarrhea, *B. bifidum* and *B. animalis*.

We interpret the results of COH IRB 12096 cautiously, as there are several caveats to this work:

1. The sample size is small,
2. There was heterogeneity of treatments rendered
3. Stool collections were done at random time points
4. There was no control of dietary factors

Despite the aforementioned limitations, COH IRB 12096 established the feasibility of stool collections and subsequent bacteriomic analysis and suggested the possibility that the propensity for the diarrhea observed could be due to the low abundance of Bifidobacterium spp observed in this patient population and that supplementing with Bifidobacterium would change the flora and also change the relative abundance of Bacteroides. An SOP for stool collection and analysis was developed that will be implemented in the current study.

Psychosocial aspects

The management of psychosocial symptoms (anxiety and depression) has gained attention in cancer care. In this scenario, there is evidence showing the association between psychosocial and physical symptoms.[13]

Gastrointestinal symptoms, particularly diarrhea, is one of the common side effects among cancer patients who are receiving sunitinib therapy[3], and it is known that mood could be influenced by gastrointenstinal manifestations and biological mechanisms (as abnormalities in the gut-brain axis, shared genetic factors, inflammation) in gastrointestinal chronic inflammatory diseases[14-16]; however little is known about the relationship between psychosocial symptoms, bacteriomic profile and gastrointestinal toxicity in mRCC patients. Therefore, future studies should be conducted in order to understand this association.

To date, there is relatively limited data to characterize the symptoms of anxiety and depression in patients with renal cell carcinoma. There is no specific measure for assessing these symptoms in patients with mRCC, except for a recent guideline for screening, and assessment of anxiety and depressive symptoms in adults with cancer[17]. Generalized Anxiety Disorder (GAD)-7 is recommended as a good measure to assess significant symptoms of anxiety, and the Patient Health Questionnaire for Depression (PHQ)-9 of depression. Both measures are based on the Diagnostic and Statistical Manual of Mental Disorders (DSM).

The GAD-7[18] is a one-dimensional self-administered scale designed to assess the presence of the symptoms of anxiety. Score is calculated by simple addition of the answers to each item. Scores for all 7 items range from 0 (not at all) and 3 (nearly every day). Therefore, the total scores ranges from 0 and 21, and may be categorized into four severity groups: minimal (0-4), mild (5-9), moderate (10-14) and serious (14-20).Questionnaire administration time is 5 minutes.

The PHQ-9[16] focuses on the nine signs and symptoms of depression from the DSM, and has a dual-purpose, to establish a provisional disorder diagnosis and to provide a symptom severity score. High scores indicate increasing severity of depression. For a diagnosis of depression, five or more items must be scored as present more than half of the days or nearly every day. A PHQ-9 score of 5-9 indicates minimal depressive symptoms, a score of 10-14 is minor depression or major depression that is mild, a score of 15-19 is major depression, moderately severe, and a score > 20 indicates major depression, sever. Questionnaire administration time is 5 minutes.

Patients will be assessed for anxiety and depression at 2 distinct time-points: at baseline (before starting sunitinib - T1) and at week 13 (T2) to match the proposed assessments of stools and gastrointestinal toxicity.

2.2 Overview and rationale of study design

Diarrhea is a significant toxicity experienced by roughly 50% of patients treated with VEGF-TKIs. Identifying a pre-treatment biomarker associated with treatment-related diarrhea may lead to the development of a pre-treatment conditioning regimen. Data from our pilot study in metastatic renal cell carcinoma (mRCC) patients undergoing VEGF-TKI therapy demonstrated a notably low abundance of Bifidobacterium spp. The primary objective of this study will be to ascertain whether or not the simple addition of Activia™ yogurt (containing Bifidobacterium lactis DN-173 010) will reduce this dysbiosis. Changing the abundance of this species, and indirectly other species in the complex microbiota population prior to initiating therapy may decrease in VEGF-TKI related diarrhea.

Twenty patients with mRCC will be recruited prior to initiating standard of care therapy with VEGF-TKIs, and will be followed for three months of therapy for specimen and data collection. Patients will be treated according to NCCN guideline recommendations for each VEGF-TKI agent. An intervention will be performed. Specifically, patients will be randomized to either a probiotic-supplemented group (Activia™) or a probiotic-restricted group using a permuted block design to ensure the 20 patients were assigned to the two groups with 1:1 ratio. Patients in the probiotic-supplemented group will be asked to consume one 4 oz serving of Activia vanilla yogurt in the morning and one 4 oz serving of Activia vanilla yogurt in the evening daily for the 3 month study period and patients in the probiotic-restricted group will be asked to avoid any intake of yogurt or yogurt-containing foods for the evaluation period of the study.

Several stool collections will be performed. Stool samples will be collected prior to initiating VEGF-TKI treatment, one week after initiating therapy, two weeks after initiating therapy, three weeks after initiating therapy (i.e. after the first week off of therapy), three months (13 weeks) into VEGF-TKI therapy, and at the onset of diarrhea. A stool specimen at the onset of diarrhea would reflect changes in the bacteriome that could potentially drive onset of this symptom. Compliance with stool submission has not been an issue in our pilot study (IRB 12096). All stool samples will be collected by the participant at home and sent via overnight shipping to the laboratory at City of Hope. Participants will receive training on the sample collection process. Participants will also be asked to complete a diet log and stool frequency log that uses the CTCAE v4.0 definitions of diarrhea:

- Grade 1: Increase of <4 stools per day over baseline; mild increase in ostomy output compared to baseline
- Grade 2: Increase of 4 - 6 stools per day over baseline; moderate increase in ostomy output compared to baseline
- Grade 3: Increase of >=7 stools per day over baseline; incontinence;hospitalization indicated; severe increase in ostomy output compared to baseline; limiting self care ADL
- Grade 4: Life-threatening consequences;urgent intervention indicated
- Grade 5: Death

For purposes of the current study, we are interested in characterizing the incurrence of all grade diarrhea (e.g., grade 1-5 toxicity); however, the grade of diarrhea incurred will be specified.

From the serial stool samples, *Bifidobacterium spp.* levels and stool bacteriomic profile over time will be assessed and associations will be examined between patients who experience diarrhea and those who do not.

To explore potential confounding factors or associations of interest between the bacteriomic profile and the presence of diarrhea, data collection will include: intake of yogurt, general description of daily diet, prior anti-cancer therapies, concomitant medications during the study period, and response to VEGF-TKIs. These data will be collected from the participant's medical record or from the participant's daily log.

As differences in intestinal flora may be immune-mediated, pre-treatment levels of circulating T-regulatory cells, myeloid derived suppressor cells and levels of peripheral STAT3 will be compared in patients with and without treatment-related diarrhea. For these analyses, participants will provide a 10-ml blood sample at the time of three stool specimen collections (prior to initiating VEGF-TKIs, two weeks after initiating therapy, and three months into therapy).

3.0 PARTICIPANT NUMBER, COHORT CHARACTERISTICS, ELIGIBILITY, AND INVOLVEMENT

3.1 Sample number and cohort characteristics

Twenty COH metastatic renal cell carcinoma patients about to undergo treatment with VEGF-TKIs will be enrolled to a single cohort at COH.

3.2 Eligibility

Patient MRN:	Patient Initials: (L,F,M):

3.2.1 Inclusion Criteria

Participants must meet the following criteria on screening examination to be eligible to participate in the study:

___ 1. Age 18 or older

___ 2. Cytologically or pathologically verified diagnosis of RCC

___ 3. Diagnosis of RCC that is defined as metastatic by standard criteria (AJCC 7th edition, 2010)

___ 4. Planned treatment with any VEGF-TKI treatment with treatment has not yet begun

___ 5. Ability to understand and the willingness to sign a written informed consent

___ 6. Ability to read and write English

___ 7. Documented consent to participation to include the following study specific procedures:

a. Be able to provide to up to six serial stool collections at home and deliver to FedEx location that day as per standard operating procedure (Appendix A)
b. Have three 10-ml blood samples taken during a routine clinic visit
c. To not take probiotic supplements except as oriented
d. If randomized to the probiotic-supplemented group (the yogurt-based supplement Activia), be willing to comply with daily intake and record this intake as a component of a dietary log. The patient will be asked not to take any yogurt or yogurt-containing foods beyond this.
e. If randomized to the probiotic-restricted group, agree not to consume yogurt or yogurt-containing foods

Maintain a dietary log and stool frequency log (Appendix B)

3.2.2 Exclusion Criteria

Prospective participants who meet any of the following criteria will not be eligible for admission into the study:

___ 1. Patients with a known intolerance to lactose or other constituents of Activia

___ 2. Patients with irritable bowel syndrome, Crohn's disease, or other clinically significant GI related condition that might confound the VEGF-TKI-related-diarrhea endpoint

___ 3. Patients taking antibiotics or who plan to begin taking antibiotics.

___ 4. Any other condition that would, in the Investigator's judgment, contraindicate the patient's participation in the clinical study due to safety concerns or compliance with clinical study procedures (including compliance issues related to feasibility/logistics)

3.2.3 Inclusion of Women and Minorities

The study is open to all patients who meet eligibility criteria, regardless of gender or ethnicity.

3.3 Duration of Participation and Overview of What Is Expected From Participants

Participants will be in the trial for roughly three and a half months, initiating with informed consent procedures prior to commencing VEGF-TKIs and ending week 13 of VEGF-TKI therapy. Participants will provide five to six stool samples at home during participation, which they will then take to a local FedEx drop off location that same day. Participants will also provide three blood samples and complete a diet log and stool frequency log during participation. Half of all participants will be asked to consume vanilla yogurt twice a day during the study. The product is widely available and will be purchased by participant; reimbursement will be offered. Participants will be asked to refrain from taking/consuming other probiotics.

4.0 STUDY ACTIVITY TABLE

4.1 Study Activity Table

	Screening[a]	Pre-tx	VEGF-TKI treatment[b]				
			Wk 1	Wk 2	Wk3	Wk 4	Wk 13
Informed consent[c]	X						
Eligibility review[d]	X						
Registration[e]	X						
Participant orientation[f]		X[f]					
Research blood collection[g]		X[g]			X[g]		X[g]
Planned stool specimens[h]		X[i]		X[j]	X[j]	X[j]	X[j]
Stool specimen at onset of diarrhea[h]			------------------X[k] -----------------				
Study log review/collection					X[l]		X[l]
Data collection [m]			X		X		X
Dietary intervention[n]		X[n]		X[n]	X[n]	X[n]	X[n]
PHQ-9 and GAD-7 [o]		X					X

a. Screening procedures are to occur prior to research sample collection with the following caveat: If the sample is collected on the same day as completion of eligibility review, the informed consent and eligibility review must precede sample collection; registration must occur that very day but may be before or after sample collection.
b. Participants who end vascular endothelial growth factor tyrosine kinase inhibitor (VEGF-TKI) treatment prior to Week 13 will jump to the 'Week 13' assessments which will be performed within 1 week of the decision to end VEGF-TKI treatment.
c. Informed consent to occur before any study specific screening procedures.
d. Inclusion/exclusion criteria are found in Section 3.2. Source documentation providing investigator's confirmation that patient has met all eligibility criteria must be available prior to registration.
e. Registration into MIDAS.
f. Participants will receive training on completion of the diet log, stool frequency log, and stool specimen collection SOP by the clinician investigator. This education will be noted in the medical record.
g. One sample of blood for research will be collected within 7 days before start of VEGF-TKI, at Week 3 (+/- 1 week), and at Week 13. Efforts will be made to collect the sample **at the time of routine blood sample** collection. Blood will be collected into 10 mL CPT vacutainer tube, inverted slowly about 8-10 times, maintained at RT and transported to pathology laboratory, who will ensure the sample is labeled with the "COH IRB #16088", the participants RPN, and **time** and date of collection, prior to transport to the Kortylewski laboratory. The Kortylewski laboratory should be notified of planned samples via email (mkortylewski@coh.org or other designee) preferably at least a day in advance of sample collection. The sample should be brought to the Kortylewski as soon as possible for processing to begin within 4 hours of the time of collection.
h. All stools samples to be collected at home and dropped off the same day to a FedEx location prior to that day's FedEx pick-up, per Stool Collection SOP (Appendix A).
i. Pre-treatment stool samples to be collected within 7 days of start of VEGF-TKI.
j. Week 2, Week 3, Week 4, and Week 13 stool samples to be provided any day Monday to Thursday on the designated week.

k. If diarrhea is not incurred, submission of stool is not required. Sample to be collected as early as possible after the onset of diarrhea such that the sample can be collected and delivered to FedEx drop off (Monday-Thursday only).
l. Efforts will be made to review and/or collect the study log with participants on weeks 3, 6, 9, and 13 which correspond to the times when patients taking VEGF-TKI are usually seen (and assessed with CBC w/ dif and serum chemistry) after three weeks of treatment. It will not be a deviation if the visits to review/collect study logs are delayed or combined.
m. Data will be collected and entered into eCRFs from the participant provided logs and the medical record. Data to be collected within two weeks of the end of the defined study point.
n. If patients are randomized to the probiotic-supplemented group, they will be asked to purchase Activia vanilla yogurt. Patients will be asked to consume one 4 oz serving of Activia vanilla yogurt in the morning and one 4 oz serving of Activia vanilla yogurt in the evening beginning Week 2 and through Week 13.
o. Symptoms of anxiety and depression will be assessed at the baseline (before start the treatment) and at the Week 13 (Section 8.0).

5.0 CLINICAL DATA COLLECTION

5.1 Participant Daily Diary

Participants will be oriented to complete the daily study log, found in Appendix B. The log will be collected and reviewed during visits to the clinic at the end of each 3-week treatment regimen (weeks 3, 6, 9 and 13 of treatment). The following information from the diary will be entered into the study specific database for analysis:

- Diarrhea, categorized per CTCAE 4.0 diarrhea grading. Please note that the patient will be instructed to consider diarrhea to be an increase in stool frequency of < 4 over baseline. This definition is consistent with the CTCAE 4.0 terminology for grade 1 diarrhea.
- Stool specimen collection dates
- Yogurt intake, or intake of yogurt-containing foods

5.2 Data Collected from the Medical Record

The following data will be obtained from the medical record and recorded in the study specific database:

- Age at registration, gender
- Prior anti-cancer therapy
- Concurrent medications from the time of informed consent through end of participation
- VEGF-TKI dose and administration
- Smoking status
- Response status (as noted by treating investigator: PD, SD, CR, PR)

6.0 BLOOD SPECIMENS, ASSESSMENT OF TREG, STAT3 AND MDSC

6.1 Research Blood Specimen Collection and Transfer to Processing Laboratory

One 10ml CPT tube will be collected within 7 days before start of VEGF-TKI, during week 3, and during week 13 (+/- 1 week). The pre-treatment sample may be collected on the morning of initiation of therapy, so long as the sample precedes VEGF-TKI intake. Efforts will be made to collect the sample at the time of

routine blood sample collection. Blood will be collected into 10 mL CPT vacuum tube, inverted slowly about 8-10 times, maintained at room temperature.

The sample will be labeled with the date, time of collection, IRB # 16088, and the participant's unique research participant number, prior to the prompt transport to the laboratory of Dr. Marcin Korylewski for correlative analyses.

The Kortylewski laboratory should be notified of planned samples via email (mkortylewski@coh.org) or other laboratory designee preferably at least a day in advance of sample collection.

6.2 Initial Sample Processing – Isolation of PBMCs

Processing of samples will occur at in the laboratory of Dr. Marcin Kortylewski. The 10ml CPT tube samples will be processed within 4 hours of collection to avoid degradation of the phosphomoieties (i.e., pSTAT3) assessed in this protocol. CPT tubes will be centrifuged at 1800 x g (approximately 2800 rpm on a Sorvall RT6000 centrifuge) for 20 minutes at room temperature. After centrifugation, plasma in the CPT tubes will be gently pipetted against the gel plug to dislodge cells stuck to the top of the gel. The cell suspension will be transferred to a 50 mL conical polypropylene tube. cRPMI will be added to a total of 40 mL. A 10 mL aliquot of cell suspension for counting will be removed. The 50 mL tubes will then be centrifuged at 250 x g for seven minutes at room temperature. When centrifugation is complete, the supernatant will be aspirated. PMBCs will be either cryopreserved or used fresh.

6.3 Sample Analysis

Analysis of samples for Treg, STAT3 and MDSC will occur in the laboratory of Dr. Marcin Kortylewski. Relevant WBC subsets will be conducted through previously reported techniques.[19] PBMCs will be immersed in a mixture of PBS, 2% FCS and 0.1% (wt/vol) sodium azide with Fc III/IIR-specific antibody to block nonspecific binding and stained the cells with different combinations of fluorochrome-coupled antibodies to CD11c, I-A^b (MHC class II), CD86, CD11b, Gr1, CD49b, CD3, CD25 or Lag-3, or with annexin V (BD Biosciences). We collected fluorescence data on FACSCalibur (Beckton Dickinson) and analyzed them using FlowJo software (Tree Star). This method has been previously published by Chalmin *et al.*[20]

7.0 DIETARY INTERVENTION, STOOL SPECIMENS, MICROBIOME PROFILING

7.1 Dietary Intervention

Using methods described in statistical analysis, patients will be randomized either to a probiotic-supplemented group or a probiotic-restricted group for 3 months. If patients **are** randomized to the probiotic-supplemented group, they will be asked to purchase Activia vanilla yogurt. Patients will be asked to consume one 4 oz serving of Activia vanilla yogurt in the morning and one 4 oz serving of Activia vanilla yogurt in the evening for a total of 3 months. Patients will be cautioned not to take alternative Activia products, such as Activia Greek yogurt, Activia drinks, etc., and should not consume other flavors of Activia yogurt (i.e., fruit-containing yogurt). Patients will further be asked to be mindful of storage directions for Activia, as listed in the product label, and to further abide by expiry dates for the product. Patients enrolled on this arm should not take other yogurt or yogurt-containing foods, and should also refrain from probiotic supplements during the three month study period. Notably, methods for this randomization (i.e., recommendation of a dietary intervention without direct supply of actual food contents) replicates the format of a large prospective study published in the New England Journal of Medicine assessing the cardiac benefit of a Mediterranean diet.[21] If patients are randomized to the probiotic-restricted group, they will be asked to not take other yogurt or yogurt-containing foods, and should also refrain from probiotic supplement during the 3 month study period. At the conclusion of the 3-month study period (or earlier if VEGF-TKIis withdrawn due to progression or toxicity or the patient is taken off this protocol

by patient or physician choice), patients may submit receipts to the study coordinator for reimbursement for Activia. As discussed subsequently, the dietary logs should reflect all oral intake, including dietary interventions prescribed by this study.

7.2 Stool Specimen Collection

Fecal material will be collected in a 100 mL collection container by patients. A standard operating procedure (SOP) has been generated for stool collection, as outlined in Appendix A. Stool collection kit contents are listed in Appendix B. A copy of this SOP will be provided to the patient and their understanding of the SOP will be documented by the PI.

All samples will be collected by participants at home and dropped to a FedEx location on the day of sample collection and prior to that day's final delivery. Samples will be affixed with the subject's research participant number and the date on which the sample was collected. The collection container will be placed in a FedEx box that is pre-labeled with the address of Dr. Ravi Salgia's laboratory. The sender address will be denoted as the City of Hope Duarte campus (1500 E. Duarte Rd) and will not show the subject's personal address. The sample will arrive at Dr. Ravi Salgia's laboratory the next day and will be stored in Dr. Ravi Salgia's laboratory until processing and analysis.

Samples will be collected pre-treatment, week 2, week 3, week 4, and week 13 of VEGF-TKI treatment. Participants who stop taking VEGF-TKI prior to week 13 will have a final sample collected within a week of the decision to end VEGF-TKI treatment.

In addition, participants will be requested to provide a stool sample at the earliest feasible occasion following onset of diarrhea. The specimen should be collected and dropped off to a FedEx location prior to that day's final pickup on a Monday through Thursday.

7.3 Laboratory Processing and Analysis

All stool samples from subjects accrued to the study on or before the the date of this protocol will be sent to and stored in the laboratory of Dr. Ravi Salgia. Stool stored in Dr. Ravi Salgia's laboratory will be transferred to Dr. Sarah K. Highlander's laboratory at TGen North, to the attention of John Gillece (3051 W. Shamrell Blvd, Suite 106, Flagstaff, AZ, 86005) for processing and final analysis. All stool samples from subjects accrued to the study after the date of this protocol will be sent directly to Dr. Sarah Highlander's laboratory at TGen North in Flagstaff, AZ.

<u>Bacterial microbiome analysis</u>

Total genomic DNA will be isolated from 0.25 g of feces using the PowerSoil DNA isolation kit (Mo Bio, USA). Purified DNA will be separated on a 1% agarose gel and quantified by densitometry and spectrophotometry (NanoDrop 1000; Thermo Scientific, USA). As described by Stearns *et al*, a PCR protocol will be used to amplify bacterial 16S rRNA genes from all samples.[8] Following PCR primers including Illumina part of adapter sequences will be used to amplify V4 and V5 regions.

V4-F: ACACTCTTTCCCTACACGACGCTCTTCCGATCTGTGCCAGCMGCCGCGGTAA.

V4-R: ACACTCTTTCCCTACACGACGCTCTTCCGATCTGTGCCAGCMGCCGCGGTAA.

V5-F: ACACTCTTTCCCTACACGACGCTCTTCCGATCTGATTAGATACCCTGGTAG.

V5-R: GTGACTGGAGTTCAGACGTGTGCTCTTCCGATCTCCGTCAATTCMTTTGAGTTT.

Complete Illumina adapter and barcodes will be added by another 5 cycles of PCR to make Illumina library. After bioanalyzer and qPCR checking for QC. Multiple libraries will be mixed equally. Paired-end of sequencing (250bp or 300bp) will be performed by Illumina MiSeq V2/V3. Sequences will be clustered at

a variety of percent identities using USEARCHalgorithm against the closed=reference.[22] Taxonomy will then be assigned using the RDP 2.4 classifier as described in *Smith et .al*.

Illumina MiSeq high throughput sequencing will be used to sequence the 16S rRNA gene. Libraries will be constructed for all samples by amplification of V4 or V5 region of bacterial 16S rRNA. Barcodes will be created to uniquely index and label each sample for multiplex Illumina sequencing with paired end reads. Multiple test runs will be created first to ensure the validity in 16S rRNA amplification from the fecal samples and standard QC will be performed to examine the quality of multiplex Illumina sequencing. Low-quality reads will be removed and only reads that are perfectly match the assembly will be kept for further downstream analysis.

Fungal microbiome analysis

DNA will be extracted from stool samples using the MagMax PowerMicrobiome extraction kit using the KingFisher Flex magnetic purification system (ThermoFisher, Waltham, MA). All DNAs will be validated for purity and integrity by agarose gel electrophoresis, and fungal load will be quantitated using the FungiQuant TaqMan assay before proceeding to sequencing. DNAs will be subjected to internally transcribed spacer gene sequencing using the)ITS4-Fun AGCCTCCGCTTATTGATATGCTTAART and 5.8S-Fun AACTTTYRRCAAYGGATCWCT primers with dual-indexed bar codes as described by Kozich et al.[14-16] A DNA mock community that contains two fungal species will be sequenced as a positive control (Zymo, D6306) as will an extraction blank as a negative control. Libraries will be quantitated using KAPA Library Quantification Kit (KAPA Biosystems), normalized and pooled then sequenced on the Illumina MiSeq instrument using the v2 kit (2 x250 bp) targeting 20,000 reads per sample.

Demultiplexed ITS reads will be processed and clustered into operational taxonomic units (OTUs) using QIIME2.[17] Demultiplexed reads will be denoised using dada2 then clustered into OTUs using q2-dbotu. Taxonomic classification will be performed using feature-classifier classify-sklearn. Various QIIME2 plugins will be used to build a phylogenetic tree (phylogeny), calculate alpha diversity within samples (diversity plugin) and beta diversity between samples (diversity plugin). We have found that simple alpha diversity calculations, such as inverse Simpson diversity, Shannon and Chao estimates, and examination of the dominant genera in each sample provides a good first impression of the composition of the sample. We will use several different types of distance methods (Bray Curtis, UniFrac) to examine and compare the community distribution of the samples. These data will be examined by multidimensional scaling or principal components analysis and heatmaps will be created using hclust2.

Whole metagenome sequencing provides a complete picture of the taxonomic composition of a microbiome than does 16S rRNA gene or ITS sequencing and also permits predictions of microbiome function, which we can tie to metabolic and proteomic results. Metagenomics also has been used for the discovery of clinical biomarkers and will allow us to identify antibiotic resistance genes. Metagenomic DNA will be sequenced on the Illumina NextSeq platform to a depth of 2 Gb/sample.[18-19] Human reads will be identified by mapping them to the human genome GRCh38.p7 using BowTie2 and they will be removed. Demultiplexed reads will be quality trimmed using Trimmomatic to remove adapters and low-quality bases and reads.[20-21] Trimmed metagenomic reads will be taxonomically profiled using MetaPhlAn 2.0. Functional profiling of the metagenomes will be performed using HUMAnN2, which annotates open reading frames (ORFs) and generates gene family abundances, metabolic pathway coverage and abundances. Antimicrobial resistance genes will be identified by mapping to a curated version of the Resfams database. Resistance genes of interest will be verified by targeted amplicon sequencing.[22-25]

8.0 STATISTICAL CONSIDERATIONS

8.1 Study Design and Statistical Analysis

Primary Objective:

Compare the change from baseline *Bifidobacterium spp.* between the Activia group and the group without Activia.

The primary measurement is the abundance of Bifidobacterium spp. in stool since probiotic formulations including *Bifidobacterium spp.* have been noted to improve acute diarrhea in a multitude of settings[11,12] and mRCC patients in our pilot study have very low abundance of Bifidobacterium spp. The objective is to determine whether the levels of *Bifidobacterium spp.* increase in patients getting Activia™, and compare to a control group. Operational taxonomic units (OTUs) for bacterial will be identified by QIIME and the relative abundance will be estimated as the percentage of reads belong to certain OUT. T-test or mann-whitney test will be used to test the difference in change from baseline in relative abundance for *Bifidobacterium spp*. between patients with or without Activia™ added to the diet. If the data appear symmetrical and the t-test is used, with 20 patients enrolled and 10 in each group, assuming equal variance in each group, using one-sided t-test at type I error of 0.05, we will have 80% power to detect a larger change in average abundance of Bifidobacterium spp in patients receiving Activia™ than those who are not randomized to take Activia™. If abundance and change is too low in the non-Activia™ arm to employ the t-test, we will employ the Mann-Whitney test. A sample size of 10 in each group will have 85% power to detect a probability of 85% that an observation in the change in Bifidobacterium spp abundance in the Activia ™ arm is larger than an observation in the change in the non-Activia ™ arm using a Wilcoxon (Mann-Whitney) rank-sum test with a 0.050 one-sided significance level.

Secondary Objectives:

1) To determine if the Activia™ reduces the incidence of diarrhea for mRCC patients treated with VEGF-TKIs.

We will compare the rate of all grade diarrhea in patients who receive the dietary supplement and those who do note. The rate of grade 3/4 diarrhea will also be compared between the two groups. A Fisher's exact test with a 0.050 one-sided significance level will have 80% power to detect the difference between the Activia™ group proportion with diarrhea, $\square_1$, of 0.200 and a non-Activia™ proportion, $\square_2$, of 0.800 when the sample size in each group is 10.

2) To compare pre-treatment levels of circulating Treg cells and levels of peripheral STAT3 in patients with and without diarrhea with sunitinib therapy, and correlate with Bifidobacterium, other bacteria, and Activia™:

Treg quantity is a secondary measurement and an analysis will be carried out to evaluate whether Tregs expression differs in patients who develop diarrhea with VEGF-TKI therapy as compared to patients who do not. The distribution of the Treg quantity will be examined and log transformation may be applied if the distribution is skewed. Parametric or nonparametric test will be used to compare Tregs between the the diarrhea and non-diarrhea groups, and between the Activia™ and non-Activia™ groups.

3) To determine if patients with the diarrhea in mRCC patients treated with VEGF-TKI therapy have a lower baseline level of *Bifidobacterium spp.*

Once diarrhea has occurred, the Bifidobacterium spp may be reduced. We will compare the abundance of Bifidobacterium spp. Between diarrhea and non-diarrhea patients and the change in Bifidobacteria in patients who subsequently get diarrhea.

4) **To explore association between psychosocial symptoms (anxiety and depression) and bacteriomic profile and gastrointestinal toxicity in mRCC patients treated with VEGF-TKIs.** We will dichotomize the population based on low or high depression (and subsequently low and high anxiety). Using descriptive statistics, we will then assess differences in response rates, gastrointesntinal toxicity, microbial diversity and immune biomarkers.

Other endpoints:

Clustering short read sequences, identify operational taxomomic units (OTUs) and summarize microbiome profiles.

QIIME will be used to cluster V5-16S rRNA Solexa reads for each sample, at the level of 97% nucleotide sequence identity. A closed-reference OUT picking protocol with USEARCH against Greengenes database will be used. Taxonomy will be assigned using RDP classifier 2.4. The relative abundance of OTUs will be calculated at phylum-, class-, order-genus- and species-level taxa. Weighted and unweighted UniFrac distances between samples will be calculated with a *de novo* tree constructed from representative sequences defining each OTU. A table of OTU counts per will be generated and used in combination with the tree to calculate beta diversity. The association between the subclusters and phenotypic variables will be studied. Principle coordinate analysis (PCoA) will be used to examine possible correlation between phylum/genus and other phenotypic variables.

.

Asssess the feasibility of stool collection and bacteriomic profiling.

We will assess the feasibility of bacteriomic profiling. Specifically, we will make note of those stool specimens for could not be performed. Reasons for an inability to perform this analysis will be noted (for instance, insufficient DNA collection) and the number of specimens that could not be analyzed will be expressed as a proportion of the overall specimens collected. These data will be integral for the future directions of this project, where stool collection will be performed for the same purposes in larger cohorts.

8.2 Sample Size and Projected Accrual Rate and Study Duration

Twenty participants will be metastatic renal cell carcinoma will enroll to the study. Enrollment is expected to be completed in 3 years, and the study is expected to be completed in 3.5 years.

9.0 HUMAN SUBJECT ISSUES

9.1 Recruitment of Subjects

Potential participants will be approached by their physicians or a member of the clinical staff and invited to participate within the outpatient clinic environment.

9.2 Advertisements

No publicly displayed advertisements will be used to recruit potential study participants.

9.3 Informed Consent

All participants will undergo standard written informed consent procedures as dictated by the City of Hope Human Research Protections Office prior to performing any screening procedures that are not part of standard-of-care. Informed consent will be obtained by the principal investigator, collaborating

investigators, or other IRB designated personnel who will meet the training requirements established by the IRB. With the support of research personnel, he/she will explain the nature, duration, purpose of the study, potential risks, alternatives and potential benefits, and all other information contained in the informed consent document. In addition, they will review the experimental subject's bill of rights and the HIPAA research authorization form. Prospective research participants will be informed that they may withdraw from the study at any time and for any reason without prejudice. Prospective research participants will be afforded sufficient time to consider whether or not to participate in the research.

9.4 Participant Withdrawal from Research

At any time after signing the informed consent document, participants may elect to withdraw from future study procedures (stool sample) but permit clinical data collection to continue, or may elect to withdraw completely from the study whereby no additional samples or data will be collected. In this later instance, data and samples already collected will continue to be used as this information will have been de-identified with the exception of the RPN. Participant withdrawal, including the nature of withdrawal, will be documented by the investigator as per standard COH practice.

9.5 Study location and Performance Sites

Participant involvement and data collection and blood sample analysis will be performed at City of Hope. Stool sample processing and analysis will be performed in Dr. Sarah K. Highlander's laboratory at TGen North in Flagstaff, AZ.

9.6 Potential Risks and Benefits to Participation

9.6.1 Potential risks to participation

The risks and discomforts of this study are minimal. The risks of drawing blood include temporary discomfort from the needle stick, minor bleeding, bruising, anemia, dizziness, and very rarely, an infection where the needle was inserted. All blood specimens requested for this study will be collected at the time of routine procedures, as such there will not be any additional needle stick, and the risk to the participant will be minimized.

The risks of establishing a data specimen repository and medical record data collection are minimal and include a possible breach of confidentiality. The risk of breach in confidentiality will be mitigated by following procedures detailed in Section 9.10.

The risks of a dietary intervention with a yogurt product are minimal and include allergic reactions (if not previously known) and minor GI-related complaints.

Furthermore, the anxiety and depression assessment have a potential risk of a temporary emotional discomfort.

9.6.2 Potential benefits to participation

There is no direct significant benefit to the research participant. However, the participant may benefit from the knowledge that his/her participation may help others.

9.6.3 Potential benefits to others

Information from this study is expected to contribute to the identification of biomarkers that may help inform treatment decisions by clinicians treating patients for cancer for which one treatment option involves a VEGF-TKI agent.

9.7 Alternatives to Participation

The patient can choose not to participate, and this will not impact their medical care in any way.

9.8 Financial Obligations and Compensation

In the event of physical injury to a research participant, resulting from research procedures, appropriate medical treatment will be available at the City of Hope to the injured research participant, however, financial compensation will not be available.

The research participant will not be paid for taking part in this study. If randomized to the probiotic-supplemented group, reimbursement will be provided for this intervention if requested by the patient.

9.9 Registration into MIDAS

Research participants will be registered into MIDAS for this trial.

9.10 Confidentiality

This research will be conducted in compliance with federal and state of California requirements relating to protected health information (PHI).

All samples will be coded prior to submission to the research laboratories. The coded identifier will be the COH research patient number (RPN), provided by the MIDAS system, which is devoid of direct participant identifiers. The key to the code is maintained in MIDAS which is a secure environment.

All study related forms including consent documents and patient pill diaries will be stored in locked and secure locations.

Medical records of participants will be securely maintained in the strictest confidence, according to current legal requirements. All information will be treated confidentially. No identifiers will be used in any subsequent publication of these results.

10.0 DATA AND SAFETY MONITORING, UNANTICIPATED PROBLEMS AND ADVERSE EVENT REPORTING

10.1 Definition of Risk Level

This is a Risk Level 2 study, as defined in the City of Hope Institutional Data and Safety Monitoring Plan [policy effective date: 07/09/14], because it involves blood sample collection, establishment of a data specimen repository, medical record data collection, and a dietary intervention with yogurt where the risk of harm is low.

10.2 Monitoring and Personnel Responsible for Monitoring

The Principal Investigator (PI) is responsible for monitoring protocol conduct and reporting to the City of Hope (COH) Data and Safety Monitoring Committee (DSMC) and Institutional Review Board (IRB) as indicated in the sections below.

10.3 Unanticipated Problems (UP) Involving Risks to Subjects or Others

An unanticipated problem is any incident, experience or outcome that meets all three of the following criteria:

1. Unexpected (in terms of nature, severity, or frequency) given the following: a) the research procedures that are described in the protocol-related documents such as the IRB approved research protocol, informed consent document or Investigator Brochure (IB); and b) the characteristics of the subject population being studied; AND
2. Related or possibly related to participation in the research (possibly related means there is a reasonable possibility that the incident, experience, or outcomes may have been caused by the procedures involved in the research); AND

3. Suggests that the research places participants or others at greater risk of harm (including physical, psychological, economic, or social harm) than previously known or recognized.

Any UP that occurs during the study conduct will be reported to the DSMC and IRB in accordance with the City of Hope's Institutional policy [policy effective date: 05/14/14] using the electronic submission system, iRIS.

10.4 Deviations

A deviation is a divergence from a specific element of a protocol and that occurred without prior IRB approval. Deviations from the approved protocol should be avoided, except when necessary to eliminate an immediate hazard to a research participant. A Corrective and Preventative Action (CAPA) plan should be developed by the study staff and implemented promptly to avoid similar issues in the future. All deviations from the protocol must be documented in study source documents and promptly reported to the DSMC and IRB.

10.5 Reporting Deviations

Investigators may deviate from the protocol to eliminate immediate hazards for the protection, safety, and well-being of the study subjects without prior IRB approval. For any such deviation, the PI will notify the DSMC and IRB, within 5 calendar days of its occurrence by electronic submission of a Deviation Notice via iRIS.

10.6 Single Subject Exception (SSE) Amendment Request

Deviations from the written protocol that are not done to eliminate an immediate hazard(s) for the protection, safety and well-being of study subjects but may increase risk and/or alter the protocol integrity require prior IRB approval. The deviation is submitted as a Single Subject Exception (SSE) amendment request. An IRB approved SSE does not need to be submitted as a protocol deviation to the DSMC. The SSE should be submitted according to the IRB guidelines and COH Institutional Deviation Policy [policy effective date: 11/07/11] and submitted via iRIS.

A deviation that is not an SSE (i.e., discovered after the occurrence) must be reported to the COH DSMC and IRB according to the COH Institutional Deviation Policy [policy effective date: 11/07/11] and submitted via iRIS.

11.0 INSTITUTIONAL REVIEW BOARD

11.1 Institutional Review Board

In accordance with City of Hope policies, an Institutional Review Board (IRB) that complies with the federal regulations at 45 CFR 46 and 21 CFR 50, 56 and State of California Health and Safety code, Title 17, must review and approve this protocol and the informed consent form and process prior to initiation of the study. All institutional, NCI, Federal, and State of California regulations must be fulfilled.

This study must be approved by an appropriate institutional review committee as defined by Federal Regulatory Guidelines (Ref. Federal Register Vol. 46, No. 17, January 27, 1981, Part 56) and the Office for Protection from Research Risks Reports: Protection of Human Subjects (Code of Federal Regulations 45 CFR 46).

12.0 REFERENCES

1. Pal SK, Figlin RA. Renal cell carcinoma therapy in 2010: many options with little comparative data. Clin Adv Hematol Oncol 2010;8:191-200.

2. Greaves TS, Olvera M, Florentine BD, et al. Follicular lesions of thyroid: a 5-year fine-needle aspiration experience. Cancer 2000;90:335-41.

3. Jonasch E, Signorovitch JE, Lin PL, et al. Treatment patterns in metastatic renal cell carcinoma: a retrospective review of medical records from US community oncology practices. Current Medical Research and Opinion 2014;30:2041-50.

4. Motzer RJ, Hutson TE, Tomczak P, et al. Overall Survival and Updated Results for Sunitinib Compared With Interferon Alfa in Patients With Metastatic Renal Cell Carcinoma. J Clin Oncol 2009;27:3584-90.

5. Rini BI, Halabi S, Rosenberg JE, et al. Phase III Trial of Bevacizumab Plus Interferon Alfa Versus Interferon Alfa Monotherapy in Patients With Metastatic Renal Cell Carcinoma: Final Results of CALGB 90206. J Clin Oncol 2010;28:2137-43.

6. Escudier BJ, Bellmunt J, Negrier S, et al. Final results of the phase III, randomized, double-blind AVOREN trial of first-line bevacizumab (BEV) + interferon-{alpha}2a (IFN) in metastatic renal cell carcinoma (mRCC). J Clin Oncol (Meeting Abstracts) 2009;27:5020-.

7. Rini BI, Escudier B, Tomczak P, et al. Comparative effectiveness of axitinib versus sorafenib in advanced renal cell carcinoma (AXIS): a randomised phase 3 trial. Lancet 2011;378:1931-9.

8. Nosov D, Bhargava P, Esteves WB, et al. Final analysis of the phase II randomized discontinuation trial (RDT) of tivozanib (AV-951) versus placebo in patients with renal cell carcinoma (RCC). ASCO Meeting Abstracts 2011;29:4550.

9. Xin H, Zhang C, Herrmann A, Du Y, Figlin R, Yu H. Sunitinib inhibition of Stat3 induces renal cell carcinoma tumor cell apoptosis and reduces immunosuppressive cells. Cancer Res 2009;69:2506-13.

10. Albiges L, Antoun S, Martin L, et al. Effect of everolimus therapy on skeletal muscle wasting in patients with metastatic renal cell carcinoma (mRCC): Results from a placebo-controlled study. ASCO Meeting Abstracts 2011;29:319.

11. NCT01265901: A Randomized, Controlled Phase III Study Investigating IMA901 Multipeptide Cancer Vaccine in Patients Receiving Sunitinib as First-line Therapy for Advanced/Metastatic Renal Cell Carcinoma (Available at http://www.clinicaltrials.gov; last accessed Augusts 15, 2011.).

12. Agata N, Ueno N, Houzawa H, et al. Interim safety results from the all cases post-marketing study (PMS) of sunitinib in 1,027 Japanese patients with renal cell carcinoma (RCC) or gastrointestinal stromal tumor (GIST). ASCO Meeting Abstracts 2011;29:e15133.

13. Angevin E, Grunwald V, Ravaud A, et al. A phase II study of dovitinib (TKI258), an FGFR- and VEGFR-inhibitor, in patients with advanced or metastatic renal cell cancer (mRCC). ASCO Meeting Abstracts 2011;29:4551.

14. Liu CM, Kachur S, Dwan MG, Abraham AG, Aziz M, Hsueh PR, Huang YT, Busch JD, Lamit LJ, Gehring CA et al: FungiQuant: a broad-coverage fungal quantitative real-time PCR assay. BMC Microbiol 2012, 12:255.

15. Taylor DL, Walters WA, Lennon NJ, Bochicchio J, Krohn A, Caporaso JG, Pennanen T: Accurate Estimation of Fungal Diversity and Abundance through Improved Lineage-Specific Primers Optimized for Illumina Amplicon Sequencing. Appl Environ Microbiol 2016, 82(24):7217-7226.

16. Kozich JJ, Westcott SL, Baxter NT, Highlander SK, Schloss PD: Development of a dual-index sequencing strategy and curation pipeline for analyzing amplicon sequence data on the MiSeq Illumina sequencing platform. Appl Environ Microbiol 2013, 79(17):5112-5120.

17. Bolyen E, Rideout JR, Dillon MR, Bokulich NA, Abnet CC, Al-Ghalith GA, Alexander H, Alm EJ, Arumugam M, Asnicar F et al: Reproducible, interactive, scalable and extensible microbiome data science using QIIME 2. Nat Biotechnol 2019, 37(8):852-857.

18. Wen C, Zheng Z, Shao T, Liu L, Xie Z, Le Chatelier E, He Z, Zhong W, Fan Y, Zhang L et al: Quantitative metagenomics reveals unique gut microbiome biomarkers in ankylosing spondylitis. Genome Biol 2017, 18(1):142.

19. Yu J, Feng Q, Wong SH, Zhang D, Liang QY, Qin Y, Tang L, Zhao H, Stenvang J, Li Y et al: Metagenomic analysis of faecal microbiome as a tool towards targeted non-invasive biomarkers for colorectal cancer. Gut 2017, 66(1):70-78.

20. Langmead B, Salzberg SL: Fast gapped-read alignment with Bowtie 2. Nat Methods 2012, 9(4):357-359.

21. Bolger AM, Lohse M, Usadel B: Trimmomatic: a flexible trimmer for Illumina sequence data. Bioinformatics 2014, 30(15):2114-2120.

22. Segata N, Waldron L, Ballarini A, Narasimhan V, Jousson O, Huttenhower C: Metagenomic microbial community profiling using unique clade-specific marker genes. Nat Methods 2012, 9(8):811-814.

23. Truong DT, Franzosa EA, Tickle TL, Scholz M, Weingart G, Pasolli E, Tett A, Huttenhower C, Segata N: MetaPhlAn2 for enhanced metagenomic taxonomic profiling. Nat Methods 2015, 12(10):902-903.

24. Franzosa EA, McIver LJ, Rahnavard G, Thompson LR, Schirmer M, Weingart G, Lipson KS, Knight R, Caporaso JG, Segata N et al: Species-level functional profiling of metagenomes and metatranscriptomes. Nat Methods 2018, 15(11):962-968.

25. Gibson MK, Forsberg KJ, Dantas G: Improved annotation of antibiotic resistance determinants reveals microbial resistomes cluster by ecology. ISME J 2015, 9(1):207-216.

APPENDIX A: STOOL COLLECTION PROCEDURE

STOOL COLLECTION KIT GENERAL INSTRUCTIONS

As a part of your participation in the current study, we have some specific instructions related to collection of stool. Please abide by these instructions, as they are essential for the proper conduct of the study.

Keep a "cold pack" in your freezer so that it is ready when it is needed. (We suggest putting them in your freezer when you get home).

Samples should be collected only if:

- It is a Monday, Tuesday, Wednesday or Thursday.
- They can be taken to the FedEx drop-off location before the last pick-up time.

The last pick-up time is different for different locations; it can be as early as 3:00PM.

Ask a study team member if you need assistance to locate a FedEx drop-off location or find the time of the last pick-up.

You are being asked to collect samples at the following times:

- Before you begin treatment with VEGF-TKI :___________
- The week after the week you started VEGF-TKI:__________
- Two weeks after you started VEGF-TKI:____________
- Three weeks after you started VEGF-TKI: ___________
- The thirteenth week after you started treatment with VEGF-TKI:________________
- On the first day you have diarrhea and the sample can be collected and dropped off (see example below).

Examples of when to collect the diarrhea samples:

EXAMPLE 1: If diarrhea begins on a Saturday, wait until Monday to collect a sample and take it that day to the FedEx drop off. You do not need to still have diarrhea on Monday.

EXAMPLE 2: If diarrhea begins on Wednesday, but you cannot take the sample to the FedEx location that day on time, wait until Thursday (the day after start of diarrhea) to collect the sample and take it to the drop off location.

If you have any questions about sample collection, please call this number: _______________________

USING THE STOOL COLLECTION KIT

Before you begin, review the following:

a. The ice pack is cold (has been in the freezer).
b. It is a Monday, Tuesday, Wednesday or Thursday.
c. You are able to deliver the sample to the FedEx location before the final pick-up time.

**STEP ONE:** Please place the *collection hat* around the rim of your toilet seat to allow for stool collection.	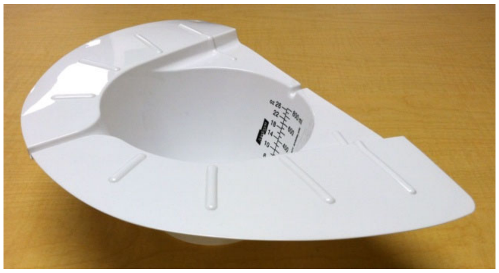
**STEP TWO:** Use the small wooden stick provided to place a small sample of stool (about the size of your thumb) in the provided plastic container.	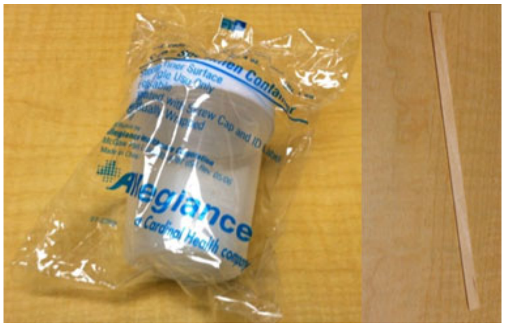
**STEP THREE:** Seal the plastic container tightly. Write today's date on the label.	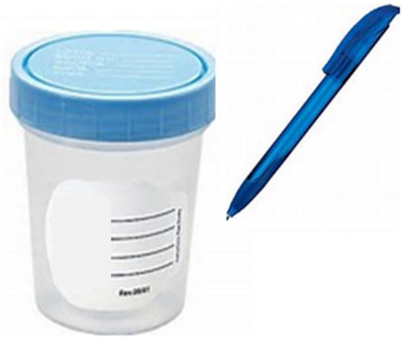
**STEP FOUR:** Place the plastic container in the bag, and seal the back using the adhesive tape already present on the bag.	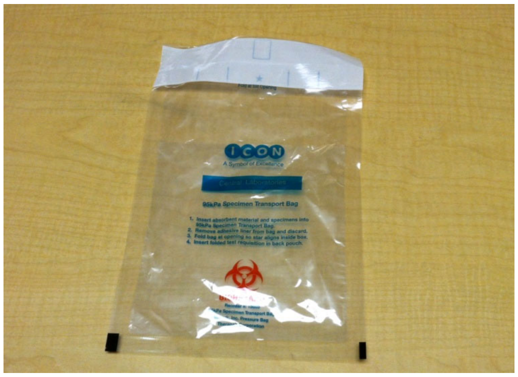

**STEP FIVE:** Wrap the bag in the "cold pack". You should have the cold pack stored in your freezer (ideally, you will do this as soon as you bring the kit home).	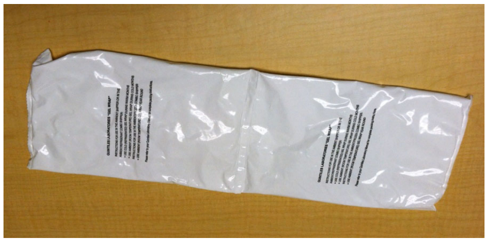
**STEP SIX:** Place the bag, surrounded by the cold pack, in the Styrofoam box.	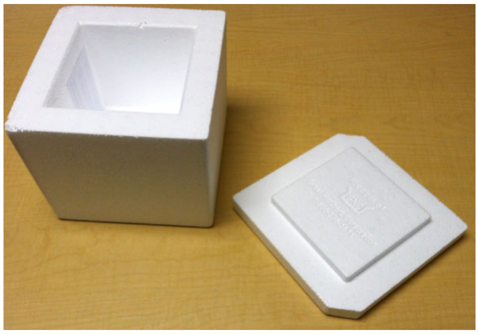
**STEP SEVEN:** Place the Styrofoam box in the cardboard box. This box should already have a mailing label affixed to it, addressed to our research group at City of Hope.	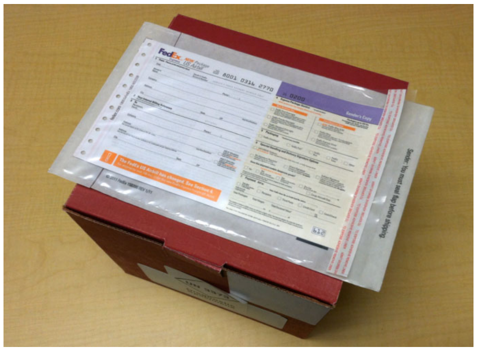
**STEP EIGHT:** Drop the box off at the nearest FedEx location this same day before the final pick-up time.	**THANK YOU FOR YOUR PARTICIPATION!**

APPENDIX B: DIET AND STOOL FREQUENCY LOG

DIET and STOOL FREQUENCY LOG - GENERAL INSTRUCTIONS

As a part of your participation in the current study, we are requesting that you complete a study log every day.

General pointers:

- When you come to the clinic, bring your logs with you.
- Each page has room for seven days – one row should be completed for each day.
- If you are randomized to Group A, please consume one 4 oz serving of Activia vanilla yogurt in the morning and one 4 oz serving of Activia vanilla yogurt in the evening. Please **do not** take any more yogurt or yogurt-containing foods beyond this. Please also **do not** take alternative Activia products, such as Activia Greek yogurt, Activia drinks, etc., or other flavors of Activia yogurt (i.e., fruit-containing yogurt
- If you are randomized Group B, please **avoid any intake of yogurt or yogurt-containing foods**.

Example of how the top part of the log will look:

- A study team member will complete the information in this box before you leave the clinic.

COMPLETED BY STUDY TEAM	Participant Initials: JSM	Participant Research Number: 1001	Group: A

Example of how the information you enter might look:

- You or someone close to can complete the log for you, so long as the information is correct.
- List all prescription and non-prescription medications.
- The person who completes that day's entry should write his or her initials in the last column.

Day and Date	General description of food I ate:	*Did I eat yogurt or take probiotics?*	*How was my stool frequency?*	*Was a stool sample collected?*	*Medications taken*	*Initials of person filling information*
	Eggs, toast, juice<br>Ham sandwich, coke, potato chips<br>Steak, mashed potatoes, wine	○ Yes<br>☒ No	☒ Seems like baseline<br>○ 1-3 stools more than baseline<br>○ 4-6 stools more than normal<br>○ 7 or more than baseline, or incontinence	○ Yes<br>☒ No	Vitamin C, Lipitor	JBS
			○ Seems like baseline			

Example of the signature line:

- When you hand over the document to the study team, they will ask to sign and date at the bottom of each log if you agree that the information is complete and correct.

At the time of handing over the document -- Participant Signature: Joseph Black Smith Date 12/18/2002

COMPLETED BY STUDY TEAM	Participant Initials:	Participant Research Number:	Group:

Day and Date	General description of food I ate:	*Did I eat yogurt or take probiotics?*	*How was my stool frequency?*	*Was a stool sample collected?*	*Medications taken*	*Initials of person filling information*
	Eggs, toast, juice<br>Ham sandwich, coke, potato chips<br>Steak, m ashed potatoes, wine	○ Yes<br>☒ No	☒ Seems like baseline<br>○ 1-3 stools more than baseline<br>○ 4-6 stools more than normal<br>○ 7 or more than baseline, or incontinence	○ Yes<br>☒ No	Vitamin C, lipitor	JBC
		○ Yes<br>○ No	○ Seems like baseline<br>○ 1-3 stools more than baseline<br>○ 4-6 stools more than normal<br>○ 7 or more than baseline, or incontinence	○ Yes<br>○ No		
		○ Yes<br>○ No	○ Seems like baseline<br>○ 1-3 stools more than baseline<br>○ 4-6 stools more than normal<br>○ 7 or more than baseline, or incontinence	○ Yes<br>○ No		
		○ Yes<br>○ No	○ Seems like baseline<br>○ 1-3 stools more than baseline<br>○ 4-6 stools more than normal<br>○ 7 or more than baseline, or incontinence	○ Yes<br>○ No		
		○ Yes<br>○ No	○ Seems like baseline<br>○ 1-3 stools more than baseline<br>○ 4-6 stools more than normal<br>○ 7 or more than baseline, or incontinence	○ Yes<br>○ No		
		○ Yes<br>○ No	○ Seems like baseline<br>○ 1-3 stools more than baseline<br>○ 4-6 stools more than normal<br>○ 7 or more than baseline, or incontinence	○ Yes<br>○ No		

		◯ Yes<br>◯ No	◯ Seems like baseline<br>◯1-3 stools more than baseline<br>◯4-6 stools more than normal<br>◯7 or more than baseline, or incontinence	◯ Yes<br>◯ No		

At the time of handing over the document -- Participant Signature: ____ Date _____________

APPENDIX C: AT HOME SAMPLE COLLECTION KIT CONTENTS

<u>Contents</u> of each kit to be provided to participants for at home collection:

Copy of Appendix A, Instructions for Stool Specimen Collection

Stool collection hat

Specimen container

With label attached

- **Label should have participant identifier added;** the participant will be oriented to add the date himself/herself.

Plastic seal bag

Cold pack

Styrofoam box

Cardboard box (into which Styrofoam box fits)

Prepaid FedEx shipping pouch

- **Shipping label should already be attached with COH as the sender and the recipient**

APPENDIX D: YOGURT-INTAKE LOG

YOGURT-INTAKE LOG - GENERAL INSTRUCTIONS

As a part of your participation in the current study, we are requesting that you complete a yogurt-intake log every day.

General pointers:

- When you come to the clinic, bring your logs with you.
- Each page has room for seven days – one row should be completed for each day.
- You have been randomized to the probiotic-supplemented group. Please consume one 4 oz serving of Activia vanilla yogurt in the morning and one 4 oz serving of Activia vanilla yogurt in the evening. Please **do not** take any more yogurt or yogurt-containing foods beyond this. Please also **do not** take alternative Activia products, such as Activia Greek yogurt, Activia drinks, etc., or other flavors of Activia yogurt (i.e., fruit-containing yogurt).
- Please log the serving size and time of each yogurt-intake

Example of how the top part of the log will look:

- A study team member will complete the information in this box before you leave the clinic.

COMPLETED BY STUDY TEAM	Participant Initials: JSM	Participant Research Number: 1001	Group: A

Example of how the information you enter might look:

- You or someone close to can complete the log for you, so long as the information is correct.
- The person who completes that day's entry should write his or her initials in the last column.

Day and Date		*Serving Size*	*Time of Yogurt-Intake*	*Initials of person filling information*
7/1/2016	MORNING	4 OZ	10:00 AM	JTC
	EVENING	4 OZ	7:00 PM	
	MORNING			

Example of the signature line:

- When you hand over the document to the study team, they will ask to sign and date at the bottom of each log if you agree that the information is complete and correct.

COMPLETED BY STUDY TEAM	Participant Initials:	Participant Research Number:	Group:

Day and Date		***Serving Size***	***Time of Yogurt-Intake***	***Initials of person filling information***
7/1/2016	**MORNING**	4 oz	10:00 AM	JBC
	**EVENING**	4 oz	7:00 PM	
	**MORNING**			JBC
	**EVENING**			
	**MORNING**			JBC
	**EVENING**			
	**MORNING**			JBC
	**EVENING**			
	**MORNING**			JBC
	**EVENING**			
	**MORNING**			JBC
	**EVENING**			
	**MORNING**			JBC
	**EVENING**			

At the time of handing over the document -- Participant Signature: ____ Date ___________

APPENDIX E: PHQ-9

Samples questionnaire is shown below.

PATIENT HEALTH QUESTIONNAIRE (PHQ-9)

NAME:________________________ **DATE:**____________

Over the last *2 weeks,* how often have you been bothered by any of the following problems? *(use "✓" to indicate your answer)*	Not at all	Several days	More than half the days	Nearly every day
**1.** Little interest or pleasure in doing things	0	1	2	3
**2.** Feeling down, depressed, or hopeless	0	1	2	3
**3.** Trouble falling or staying asleep, or sleeping too much	0	1	2	3
**4.** Feeling tired or having little energy	0	1	2	3
**5.** Poor appetite or overeating	0	1	2	3
**6.** Feeling bad about yourself—or that you are a failure or have let yourself or your family down	0	1	2	3
**7.** Trouble concentrating on things, such as reading the newspaper or watching television	0	1	2	3
**8.** Moving or speaking so slowly that other people could have noticed. Or the opposite – being so figety or restless that you have been moving around a lot more than usual	0	1	2	3
**9.** Thoughts that you would be better off dead, or of hurting yourself	0	1	2	3
	add columns		+	+

(Healthcare professional: For interpretation of TOTAL, please refer to accompanying scoring card). TOTAL:

**10.** If you checked off *any problems,* how *difficult* have these problems made it for you to do your work, take care of things at home, or get along with other people?	
	Not difficult at all ______
	Somewhat difficult ______
	Very difficult ______
	Extremely difficult ______

Copyright © 1999 Pfizer Inc. All rights reserved. Reproduced with permission. PRIME-MD© is a trademark of Pfizer Inc.
A2663B 10-04-2005

APPENDIX E: GAD-7

Sample questionnaire is shown below

Generalized Anxiety Disorder 7-item (GAD-7) scale

Over the last 2 weeks, how often have you been bothered by the following problems?	Not at all sure	Several days	Over half the days	Nearly every day
1. Feeling nervous, anxious, or on edge	0	1	2	3
2. Not being able to stop or control worrying	0	1	2	3
3. Worrying too much about different things	0	1	2	3
4. Trouble relaxing	0	1	2	3
5. Being so restless that it's hard to sit still	0	1	2	3
6. Becoming easily annoyed or irritable	0	1	2	3
7. Feeling afraid as if something awful might happen	0	1	2	3
Add the score for each column	+	+	+	
Total Score *(add your column scores)* =				

If you checked off any problems, how difficult have these made it for you to do your work, take care of things at home, or get along with other people?

Not difficult at all __________
Somewhat difficult _________
Very difficult _____________
Extremely difficult _________

Source: Spitzer RL, Kroenke K, Williams JBW, Lowe B. A brief measure for assessing generalized anxiety disorder. *Arch Inern Med.* 2006;166:1092-1097.
